# Cupric Ions Induce the Oxidation and Trigger the Aggregation of Human Superoxide Dismutase 1

**DOI:** 10.1371/journal.pone.0065287

**Published:** 2013-06-03

**Authors:** Cheng Li, Wen-Chang Xu, Zhen-Sheng Xie, Kai Pan, Jiao Hu, Jie Chen, Dai-Wen Pang, Fu-Quan Yang, Yi Liang

**Affiliations:** 1 State Key Laboratory of Virology, College of Life Sciences, Wuhan University, Wuhan, China; 2 Laboratory of Proteomics, Institute of Biophysics, Chinese Academy of Sciences, Beijing, China; 3 College of Chemistry and Molecular Sciences, and State Key Laboratory of Virology, Wuhan University, Wuhan, China; Universidad de Granada, Spain

## Abstract

**Background:**

Amyotrophic lateral sclerosis (ALS), partly caused by the mutations and aggregation of human copper, zinc superoxide dismutase (SOD1), is a fatal degenerative disease of motor neurons. Because SOD1 is a major copper-binding protein present at relatively high concentration in motor neurons and copper can be a harmful pro-oxidant, we want to know whether aberrant copper biochemistry could underlie ALS pathogenesis. In this study, we have investigated and compared the effects of cupric ions on the aggregation of ALS-associated SOD1 mutant A4V and oxidized wild-type SOD1.

**Methodology/Principal Findings:**

As revealed by 90° light scattering, dynamic light scattering, SDS-PAGE, and atomic force microscopy, free cupric ions in solution not only induce the oxidation of either apo A4V or Zn_2_-A4V and trigger the oligomerization and aggregation of oxidized A4V under copper-mediated oxidative conditions, but also trigger the aggregation of non-oxidized form of such a pathogenic mutant. As evidenced by mass spectrometry and SDS-PAGE, Cys-111 is a primary target for oxidative modification of pathological human SOD1 mutant A4V by either excess Cu^2+^ or hydrogen peroxide. The results from isothermal titration calorimetry show that A4V possesses two sets of independent binding sites for Cu^2+^: a moderate-affinity site (10^6^ M^-1^) and a high-affinity site (10^8^ M^-1^). Furthermore, Cu^2+^ binds to wild-type SOD1 oxidized by hydrogen peroxide in a way similar to A4V, triggering the aggregation of such an oxidized form.

**Conclusions/Significance:**

We demonstrate that excess cupric ions induce the oxidation and trigger the aggregation of A4V SOD1, and suggest that Cu^2+^ plays a key role in the mechanism of aggregation of both A4V and oxidized wild-type SOD1. A plausible model for how pathological SOD1 mutants aggregate in ALS-affected motor neurons with the disruption of copper homeostasis has been provided.

## Introduction

Amyotrophic lateral sclerosis (ALS), partly caused by the mutations and aggregation of human copper, zinc superoxide dismutase (SOD1, EC 1.15.1.1), is a fatal degenerative disease of motor neurons [1]. SOD1 is a major antioxidant enzyme, catalyzing the dismutation of superoxide anion radical to hydrogen peroxide and molecular oxygen [2], [3]. So far over 150 different mutations in human SOD1 have been found in familial ALS patients [4]–[9].

Copper is the third-most abundant transition metal in the brain, with average neural copper concentrations on the order of 100 µM [10]. On the one hand, copper is a cofactor for many enzymes such as SOD1 and plays an important role in central nervous system development [10]–[13]. On the other hand, copper is too redox active to exist in an unbound form in the cell without causing oxidative damage [12]–[14], and disruption of copper homeostasis is implicated in a number of neurodegenerative diseases, including Alzheimer disease and ALS [10]–[13].

SOD1 is a major copper-binding protein present at relatively high concentration in motor neurons and copper can be a harmful pro-oxidant promoted by SOD1 mutants [9], [15]. On the one hand, Cys-111-modified mutant SOD1 induces oxidative stress through aberrant copper binding [16], and increased affinity for copper mediated by Cys-111 in forms of mutant SOD1 is linked to familial ALS [17]. Furthermore, decreasing intracellular Cu^2+^ has been found to alleviate ALS phenotype in SOD1 mutant transgenic mice [18]–[21]. On the other hand, SOD1 mutants induce motor neuron disease independent of copper incorporation into SOD1 mutants mediated by copper chaperone for SOD1 (CCS) [22], and copper-binding-site-null SOD1 still causes ALS in transgenic mice [23], [24]. Therefore, we want to know whether aberrant copper biochemistry could underlie ALS pathogenesis.

Recently, it has been reported that wild-type and mutant SOD1 share an aberrant conformation and a common pathogenic pathway in ALS [25], [26]. It have been suggested that the phenotype of sporadic ALS could be regulated by the conformational change of oxidized wild-type SOD1 [25]. The neuronal toxicity by oxidatively-modified SOD1 in ALS pathogenesis is closely related to its conformational change [9]. It has clarified a possible aberrant interaction of SOD1 mutants with Cu^2+^ outside the active site in the context of familial ALS [16], [17]. Because copper is catalytically redox-active and has a potential to oxidize SOD1 itself, inappropriate reactivity of Cu^2+^ coordinated in SOD1 could underlie either the conformational change of mutant SOD1 in familial ALS pathogenesis [9] or that of oxidized wild-type SOD1 in sporadic ALS pathogenesis.

But it remains unclear so far how SOD1 mutants gain new aberrant toxic functions through binding to Cu^2+^. Pathological human SOD1 mutant A4V is the most common familial ALS mutation in North America and has a particularly short disease duration [15], [27]. So far an upper shifted band on SDS-PAGE representing oxidized wild-type SOD1 has been observed when cupric ions are added [28]. In the present study, by using several biophysical methods, such as 90° light scattering, dynamic light scattering, atomic force microscopy (AFM), mass spectrometry (MS), size-exclusion chromatography, and isothermal titration calorimetry (ITC), we investigated and compared the effects of cupric ions on the aggregation of ALS-associated SOD1 mutant A4V and wild-type SOD1 oxidized by hydrogen peroxide. A full comparison between copper and hydrogen peroxide mediated oxidation was performed to distinguish between these two oxidative environments. Our results indicated that Cu^2+^ bound to both A4V and oxidized wild-type SOD1 *via* two independent binding sites, one with a moderate affinity and one with a high affinity, and that Cys-111 is a primary target for oxidative modification of A4V by either excess Cu^2+^ or hydrogen peroxide. Further, we demonstrated that excess cupric ions not only induced the oxidation of either apo A4V or Zn_2_-A4V and triggered the aggregation of oxidized A4V under copper-mediated oxidative conditions, but also triggered the aggregation of non-oxidized A4V, and thus provided a plausible model to explain how pathological SOD1 mutants misfold in ALS-affected motor neurons.

## Materials and Methods

### Ethics Statement

All research involving original human work was approved by the Institutional Review Board of the College of Life Sciences, Wuhan University (Wuhan, China), leaded by Dr. Hong-Bing Shu, the Dean of the college, in accordance with the guidelines for the protection of human subjects. Written informed consent for the original human work that produced the plasmid samples was obtained.

### Materials

Iodoacetamide and trypsin were purchased from Sigma-Aldrich (St. Louis, MO). All other chemicals used were made in China and were of analytical grade. Unless otherwise stated, all of the reagent solutions were prepared in 20 mM Tris-HCl buffer (pH 7.4) and cupric ions were in the form of Cu^2+^-Tris complexes.

### Plasmids and Proteins

Pathological human SOD1 mutant A4V was generated from wild-type human SOD1 which cloned in pET3d vector (kindly provided by Dr. Thomas O’Halloran) using primers CTTCAGCACGCACACGACCTTCGTGGCCATGG/CCATGGCC ACGAAGGTCGTGTGCGTGCTGAAG. Single cysteine mutant C111S was generated in a similar manner. A4V, wild-type SOD1, and C111S were expressed in *Escherichia coli* and purified to homogeneity by Q-Sepharose chromatography as described [29]. Purified human SOD1 was analyzed by SDS-PAGE with one band. The demetallated (apo) SOD1 was prepared according to previously published protocols [30]. The concentration of human SOD1 was determined according to its absorbance at 280 nm using the molar extinction coefficient value of 10,800 M^−1^ cm^−1^/dimer [30].

### Measurement of SOD1 Aggregation

To obtain oxidized wild-type SOD1, wild-type SOD1 were treated with 5 mM hydrogen peroxide for 2 h, and then the samples were dialyzed against 20 mM Tris-HCl buffer (pH 7.4) extensively to remove hydrogen peroxide. Aggregation of A4V, wild-type SOD1, and oxidized wild-type SOD1 (10 µM) incubated with 50–300 µM Cu^2+^ in 20 mM Tris-HCl buffer (pH 7.4) were measured by 90^o^ light scattering on an LS-55 luminescence spectrometer (PerkinElmer Life Sciences, Shelton, CT) at 37^o^C. The excitation and emission wavelengths both were 350 nm, and the excitation and emission slit widths were 10 nm and 3 nm, respectively [31], [32]. The preparation of the samples before the first measurement took 1 min. The samples were transferred to 1-cm thermostatted quartz fluorescence cuvettes and the kinetic experiments lasted for 1–2 h. Aggregation of A4V and oxidized wild-type SOD1 (30 µM) incubated with 150–900 µM Cu^2+^ in 20 mM Tris-HCl buffer (pH 7.4) were measured by dynamic light scattering on a Zetasizer Nano ZS ZEN3600 light-scattering spectrophotometer (Malvern Instruments, Malvern, UK) at 25^o^C. The program CONTIN was used to calculate the mean hydrodynamic radius (*R*_h_). The time-dependent appearance of light scattering intensity or *R*_h_ was found to be well described by the empirical Hill equation [33], [34]:
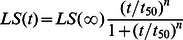
(1)

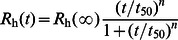
(2)where *LS*(∞) (or *R*_h_(∞)) is the light scattering intensity (or the mean hydrodynamic radius) in the long time limit, *t*_50_ is the elapsed time at which *LS* (or *R*_h_) is equal to one-half of *LS*(∞) (or *R*_h_(∞)), and *n* is a cooperativity parameter. The profile of light scattering intensity *versus* time was also analyzed as follows. A seventh order polynomial equation was fitted to all data {*t*, *LS*(*t*)} at each copper concentration. The maximum slope of the best fit polynomial (d*P*/d*t*)_max_, *k*_max_, the value of *t* corresponding to maximum slope of the polynomial t* and the value of the polynomial at this time *P*(t*) were thus evaluated [35]. In all experiments, the blanks were subtracted to correct for scattering and absorbance of buffer components.

### AFM

The formation of granular aggregates by 10 µM A4V incubated with 200 µM Cu^2+^ at various time points was confirmed by atomic force microscopy. Sample aliquots of 10 µl were deposited onto freshly cleaved mica, left on the surface for 10 min, and rinsed with H_2_O twice. Then the solution was dried in a desiccator for 12 h. AFM images were acquired in tapping mode with a SPM-9500 J3 scanning probe microscope (Shimadzu, Kyoto, Japan) [36], [37]. Several regions of the mica surface were examined to confirm that similar structures existed through the sample.

### Nano-LC-MS/MS Analysis

The upper shifted bands of 10 µM A4V incubated with either 50 µM Cu^2+^ or 5 mM hydrogen peroxide for 3 h on Coomassie Blue R250-stained SDS-PAGE were clipped out, cut into small pieces, and digested with trypsin overnight. The resultant products were centrifuged at 20,000× g for 10 min prior to analysis. The digested sample was loaded onto a homemade C18 column (100 mm × 100 µm) packed with Sunchrom packing material (SP-120-3-ODS-A, 3 µm) and followed by nano-LC-ESI-MS/MS analysis. The peptides were sequentially eluted from the high-pressure liquid chromatography (HPLC) column with a gradient of 0–90% of Buffer B (acetonitrile : water : acetic acid = 80∶ 19.9∶ 0.1, v : v : v) to Buffer A (acetonitrile : water : acetic acid = 5∶ 94.9∶ 0.1, v : v : v) at a flow rate of approximately 500 nl/min (after split) using surveyor pumps. The eluted peptides were sprayed directly from the tip of the capillary column to the LTQ mass spectrometer (ThermoFinnigan, San Jose, CA) for mass spectrometry analysis. The LTQ mass spectrometer was operated in the data-dependent mode in which first the initial MS scan recorded the mass to charge (*m*/*z*) ratios of ions over the mass range from 350–1700 Da. The five most abundant ions were automatically selected for subsequent collision-activated dissociation. All MS/MS data were searched against a human protein database downloaded from the NCBI database using the SEQUEST program (Thermo Fisher Scientific, Waltham, MA). All searches were performed using a precursor mass tolerance of 3 Da calculated using average isotopic masses. Variable modifications were set for methionine with the addition of 15.999 Da to represent methionine oxidative modification, cysteine with the addition of 57.052 Da to represent cysteine carboxyamidation, and cysteine with the addition of 32 Da or 48 Da to represent cysteine di- and tri-oxidative modification, respectively. A fragment ion mass tolerance of 1 Da was used. Enzyme cleavage specificity was set to trypsin and no more than two missed cleavages were allowed. The SEQUEST outputs were then analyzed using the commercial software Thermo Electron BioWorks (Rev. 3.3.1). The filter settings for peptides were as follows –Xcorr ≥1.9 (*z* = 1), 2.5 (*z* = 2), 3.75 (*z* = 3), Sp≥500, and Rsp≤5.

### HPLC

A TSKgel SuperSW3000 size-exclusion chromatography column (300 × 4.6 mm) was obtained from Tosoh (Tokyo, Japan). The HPLC system consisted of a LC-20AT pump and a SPD-20A UV detector (Shimadzu, Kyoto, Japan). The column was run at 37°C, with a flow-rate of 1 ml/min with a running buffer, 100 mM NaH_2_PO_4_-Na_2_HPO_4_ buffer (pH 6.7) containing 100 mM Na_2_SO_4_ and 0.05% NaN_3_. The samples of wild-type SOD1, A4V, and oxidized wild-type SOD1 were applied directly to column and eluted with the running buffer. The column was rinsed for at least 30 min at 37°C with the running buffer for the next assay.

### Isothermal Titration Calorimetry

ITC experiments on the interaction of Cu^2+^ with A4V and wild-type SOD1 oxidized by hydrogen peroxide were carried out at 25°C using an iTC_200_ titration calorimetry (MicroCal, Northampton, MA). Freshly purified A4V and freshly prepared oxidized wild-type SOD1 were dialyzed against 20 mM Tris-HCl buffer (pH 7.4) for three times at 4°C. A solution of 10.7 µM SOD1 was loaded into the sample cell (200 µl), and a solution of 250–300 µM Cu^2+^ was placed in the injection syringe (40 µl). For A4V, the first 4 injections of 1 µl of 300 µM Cu^2+^ were followed by 4 injections of 2 µl, and then 4 injections of 1 µl and 11 injections of 2 µl. For oxidized wild-type SOD1, the first injection (1 µl) of 250 µM Cu^2+^ was followed by 18 injections of 2 µl. Dilution heats of Cu^2+^ were measured by injecting Cu^2+^ solution into buffer alone and were subtracted from the experimental cures prior to data analysis. The stirring rate was 600 rpm. The resulting data were fitted to a binding model containing two sets of independent binding sites using Microcal ORIGIN Software supplied with the instrument, and the standard molar enthalpy change for the binding,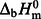, the binding constant, *K*_b_, and the binding stoichiometry, *n*, were thus obtained. The standard molar free energy change, 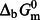, and the standard molar entropy change, 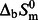, for the binding reaction were calculated by the fundamental equations of thermodynamics [33], [35], [36]:
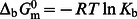
(3)

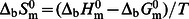
(4)


All ITC experiments were repeated three times. The experiments were pretty reproducible. Every time SOD1 (A4V and wild-type SOD1 oxidized by H_2_O_2_) possessed two sets of independent binding sites for Cu^2+^, although the thermodynamic parameters (*K*_b_, 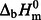 and *n*) were slightly different in different batches.

## Results

### The Presence of Cu^2+^ Triggered A4V Aggregation

**Figure 1:**
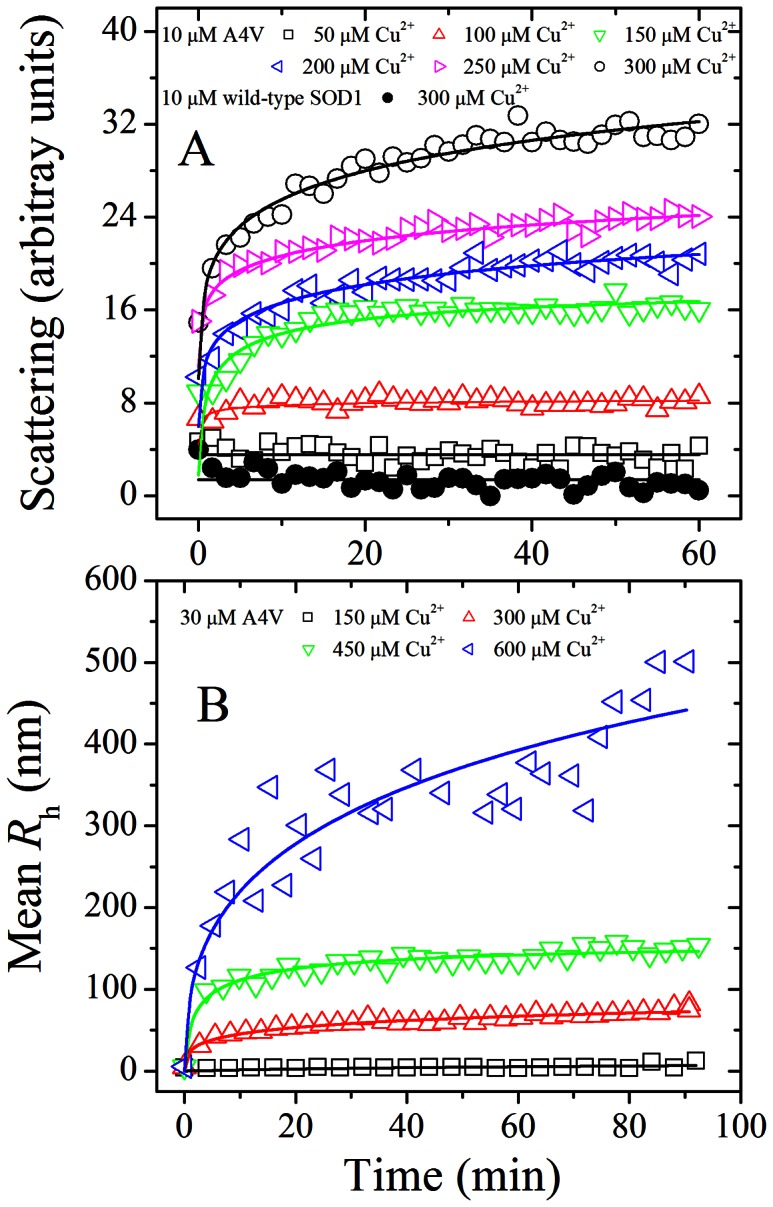
Effects of Cu^2+^ on the aggregation of pathological human SOD1 mutant. Time-course for the aggregation of A4V incubated with 50–300 µM Cu^2+^ (A) or 150–600 µM Cu^2+^ (B) in 20 mM Tris-HCl buffer (pH 7.4), compared with wild-type SOD1 incubated with 300 µM Cu^2+^ (solid circle). The final concentration of SOD1 was 10 (A) and 30 (B) µM, respectively. The copper concentrations were 50 µM (open square), 100 µM (open triangle), 150 µM (inverted open triangle), 200 µM (left open triangle), 250 µM (right open triangle), and 300 µM (open circle), respectively (A). The excitation and emission wavelengths both were 350 nm and the slit bands were 10 nm and 3 nm, respectively. The copper concentrations were 150 µM (open square), 300 µM (open triangle), 450 µM (inverted open triangle), and 600 µM (left open triangle), respectively (B). The mean hydrodynamic radius (*R*_h_) was calculated by use of the method of cumulants. The empirical Hill equation was fitted to the data and the solid lines represented the best fit. Aggregation was measured by 90^o^ light scattering at 37**°**C (A) or dynamic light scattering (B).

**Figure 2:**
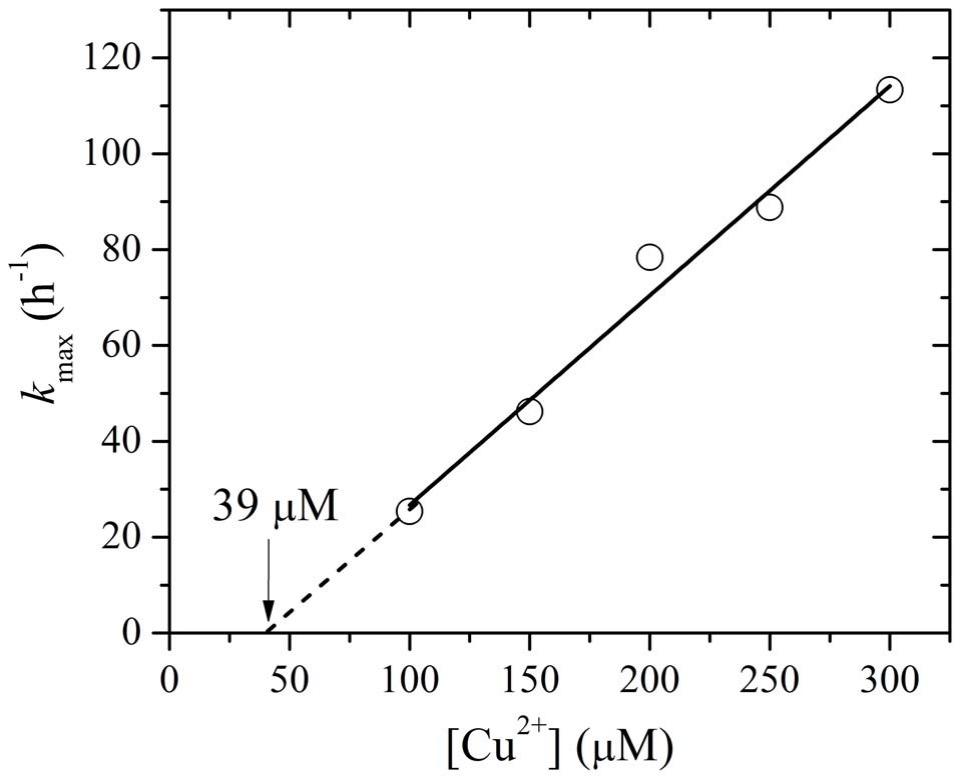
Cu^2+^-concentration dependence of the kinetic parameter *k*_max_ for the aggregation of pathological human SOD1 mutant. The data in Table 1 show a linear function for the value of *k*_max_ *versus* [Cu^2+^] for A4V with *k*_max_ = 0 at Cu^2+^ concentration of 39 µM. The data with error bars were expressed as mean ± standard errors of the mean.

90^o^ light scattering technique has been widely used for monitoring the kinetics of protein aggregation [31], [32], [38]. In this study, the effects of Cu^2+^ on the aggregation of pathological human SOD1 mutant A4V and wild-type SOD1 were examined by 90^o^ light scattering assays. An oxidative (without reducing agent present plus Cu^2+^ oxidation) experimental condition is similar to the physiological environment in ALS brain. Fig. 1A shows the time-course for the aggregation of 10 µM A4V incubated with 50–300 µM Cu^2+^ at physiological pH, compared with wild-type SOD1 incubated with 300 µM Cu^2+^. As shown in Fig. 1A, aggregation of wild-type SOD1 was not observed in the presence of 300 µM Cu^2+^ in the time scales used, but the addition of 100–300 µM Cu^2+^ did trigger A4V aggregation monitored by 90^o^ light scattering. When the concentration of Cu^2+^ went higher, the kinetic curves of A4V aggregation went up gradually, almost without a lag phase (Fig. 1A). Fitting A4V aggregation kinetic data with the empirical Hill equation (Fig. 1A) and the seventh order polynomial equation (Fig. S1A–1E) gave *LS*(∞) and *k*_max_ values, respectively, which reflect the final quantity and the elongation phase of A4V aggregation, respectively. The corresponding kinetic parameters are summarized in Table 1. As shown in Table 1, the value of *k*_max_ of A4V aggregation monitored by 90^o^ light scattering assays was 25.4, 46.2, 78.4, 88.8, and 113.4 h^−1^ in the presence of 100, 150, 200, 250, and 300 µM Cu^2+^ respectively, and the value of *LS*(∞) of A4V aggregation was 8.22, 16.36, 22.37, 26.36, and 36.72 in the presence of 100, 150, 200, 250, and 300 µM Cu^2+^ respectively. Fig. 2 shows Cu^2+^-concentration dependence of *k*_max_ for A4V aggregation. The data in Table 1 show a linear function for the value of *k*_max_ *versus* [Cu^2+^] for A4V with *k*_max_ = 0 at Cu^2+^ concentration of 39 µM (Fig. 2), suggesting that the theoretical minimum concentration of Cu^2+^ inducing 10 µM A4V aggregation should be 40 µM. Moreover, we performed parallel experiments using dynamic light scattering technique that provides quantitative estimates of the properties of SOD1 aggregate species. Fig. 1B shows the time-course for the aggregation of 30 µM A4V incubated with 150–600 µM Cu^2+^ at physiological pH. As shown in Fig. 1B, the addition of 300–600 µM Cu^2+^ did trigger 30 µM A4V aggregation monitored by dynamic light scattering. Similar to Fig. 1A, the kinetic curves of A4V aggregation went up gradually with the increase of the concentration of Cu^2+^, almost without a lag phase (Fig. 1B). Fig. S2 shows size distribution by intensity for Cu^2+^-induced SOD1 aggregation at various time points. As shown in Fig. S2, 30 µM A4V aggregated gradually when incubated with 600 µM Cu^2+^ at physiological pH. As shown in Figs. 1B and S2, the polydispersity and the average hydrodynamic radii increased with increasing both the concentration of Cu^2+^ and the incubation time. A4V SOD1 remains polydisperse upon copper addition, and becomes more polydisperse when the incubation time increases from 2.58 min to 59.34 min (Fig. S2B–2D). It should be pointed out that in our dynamic light scattering measurements all particles in the scattering volume are approximated by spheres, and such granular aggregates were observed in AFM images (see below). Therefore, as revealed by 90° light scattering and dynamic light scattering (Figs. 1 and S2 and Table 1), Cu^2+^ at a Cu^2+^/SOD1 dimer molar ratio of 10∶1 to 30∶1 did trigger the oligomerization and aggregation of A4V under copper-mediated oxidative conditions, but aggregation of wild-type SOD1 was not observed under such conditions in the time scales used.

**Table 1:** Kinetic parameters of SOD1 aggregation in the presence of different concentrations of Cu^2+^ as determined by 90^o^ light scattering assays at 37**°**C.

SOD1	[Cu^2+^] (µM)	*k*_max_ (h^−1^)	*LS*(∞)	*k*_1_ (h^−1^)	*k*_2_ (h^−1^)	*LS*_max,1_+ *LS*_max,2_
A4V	50	∼0	∼4	∼0	∼0	∼4
	100	25.4±1.1	8.22±0.05	ND	ND	8.10±0.02
	150	46.2±2.3	16.36±0.03	ND	ND	16.22±0.03
	200	78.4±1.6	22.37±0.22	34.9±8.6	4.06±0.21	20.46±0.07
	250	88.8±3.2	26.36±0.31	21.3±1.8	2.35±0.21	24.45±0.13
	300	113.4±2.7	36.72±0.51	61.8±19.0	4.11±0.13	31.67±0.08
Wild-type SOD1	300	∼0	∼2	∼0	∼0	∼2
Oxidized wild-type SOD1	150	∼0	∼3	∼0	∼0	∼3
	200	3.4±1.0	7.75±0.09	ND	ND	7.59±0.35
	250	15.1±1.5	23.47±0.25	ND	ND	24.99±0.70
	300	33.6±1.3	23.70±0.89	ND	ND	23.73±0.05

The maximum slope of the best fit polynomial (d*P*/d*t*)_max_, *k*_max_, was determined by fitting 90° light scattering intensity *versus* time to a seventh order polynomial equation. *LS*(∞) was determined by fitting 90° light scattering intensity *versus* time to the empirical Hill equation. *k*_1_, *k*_2_, *LS*_max,1_, and *LS*_max,2_ were determined by fitting 90° light scattering intensity *versus* time to a double exponential model. The final concentration of SOD1 was 10 µM. The buffer used was 20 mM Tris-HCl buffer (pH 7.4). Errors shown are standard errors of the mean.

∼, observed from the 90° light scattering curves directly.

ND, not determined because the 90° light scattering data in the present conditions could be fitted to such a double exponential model with a huge error.

### Morphology of A4V Aggregates

**Figure 3:**
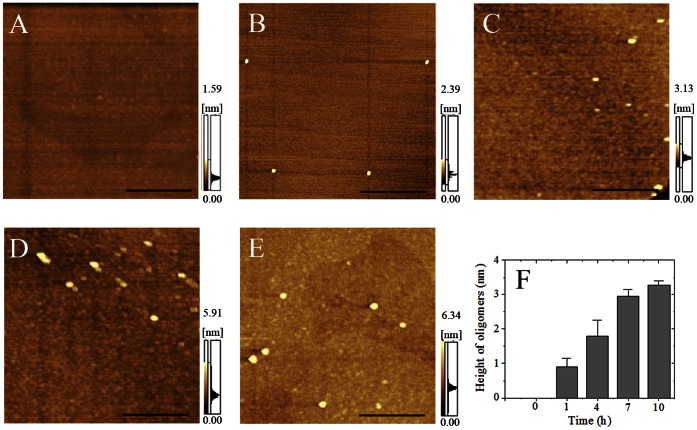
AFM images of A4V aggregates formed in the presence of copper ions. Aliquots of 10 µM A4V with 200 µM Cu^2+^ on 0 (A), 1 (B), 4 (C), 7 (D), and 10 (E) h of incubation were taken for observation with AFM, respectively. The buffer used was 20 mM Tris-HCl buffer (pH 7.4). Panel F shows the horizontal diameter at mid-height. The heights of A4V oligomers at different time points were calculated by fitting with the Guass model. The scale bars represent 100 nm.

AFM, a powerful tool for detecting the morphology of particles and aggregates [37], [39], [40], was employed to study the morphology of A4V incubated with copper. Fig. 3, A, B, C, D, and E, shows AFM images of A4V aggregates formed in the presence of 200 µM Cu^2+^ on 0, 1, 4, 7, and 10 h of incubation time, respectively. Different regions of the same sample on mica were scanned to confirm that the characteristic morphology of the sample was obtained. When 10 µM A4V was incubated with 200 µM Cu^2+^ for 1, 4, 7, and 10 h, granular aggregates with an average height of 0.91±0.24 nm (*n* = 15), 1.80±0.45 nm (*n* = 21), 2.94±0.21 nm (*n* = 19), and 3.26±0.13 nm (*n* = 25) appeared in several scanning areas, respectively (Fig. 3B–3E). With the increase of incubation time, the average height of A4V aggregates increased gradually (Fig. 3F). However, granular aggregates were not observed for 10 µM A4V incubated with 200 µM Cu^2+^ at zero time used as a control (Fig. 3A). AFM has been used to detect the morphology of fibrils/aggregates formed by proteins associated with neurodegenerative diseases such as Tau protein and prion protein, demonstrating that the heights of such fibrils/aggregates are larger than 20 nm [39], [41]–[43]. The average height of A4V aggregates we observed was much smaller than those of fibrils formed by Tau protein and prion protein [39], [41]–[43]. Our AFM results confirmed that excess cupric ions triggered the oligomerization of A4V, inducing A4V to form abundant granular aggregates under copper-mediated oxidative conditions.

### Oxidation of A4V on Cys-111 by Cu^2+^ Involved C-SO_2_H and C-SO_3_H

**Figure 4:**
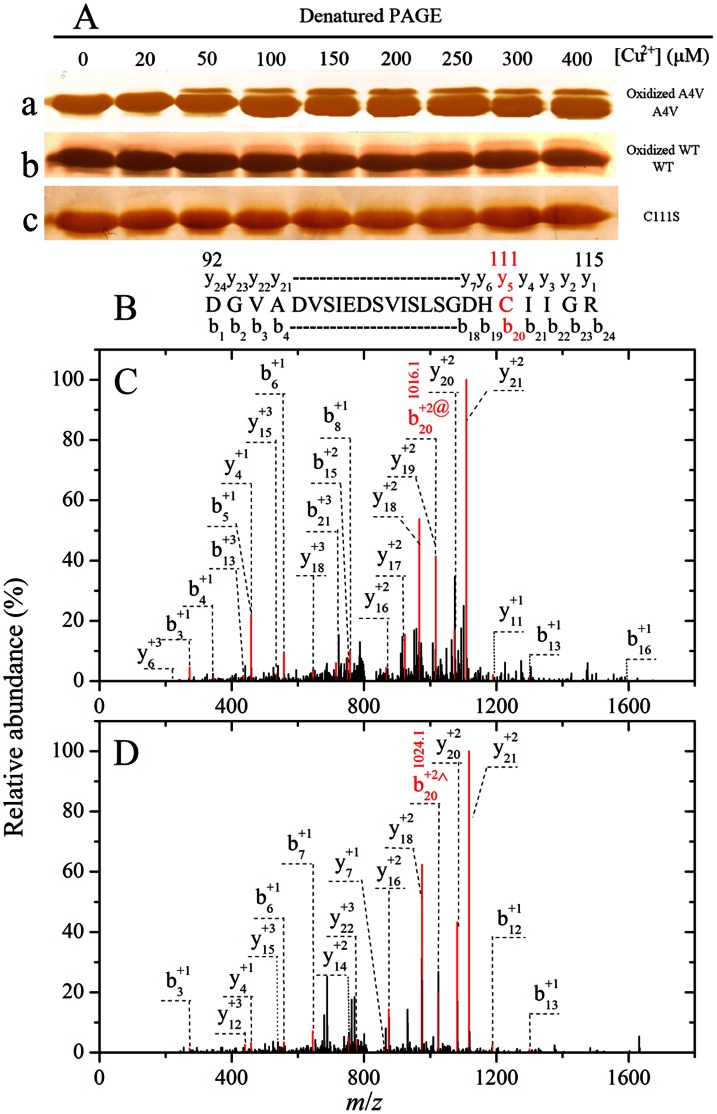
Mass spectrometry and SDS-PAGE demonstrate that Cys-111 is a primary target for oxidative modification of A4V by Cu^2+^. 13.5% SDS-PAGE of A4V (a), wild-type SOD1 (WT) (b), and C111S (c) incubated with 0–400 µM Cu^2+^ in 20 mM Tris-HCl buffer (pH 7.4) for 3 h at 37°C (A). The copper concentrations (from left to right) were 0, 20, 50, 100, 150, 200, 250, 300, and 400 µM, respectively. Gels were stained with silver. Oxidized A4V (upper shifted band) was clearly observed when A4V was incubated with 50–400 µM Cu^2+^ for 3 h (a). Schematic representation of peptide 92–115 obtained from fragmentations of A4V by LC MS/MS analysis (B). The upper shifted bands of A4V incubated with 50 µM Cu^2+^ for 3 h on Coomassie Blue R250-stained SDS-PAGE were clipped out, cut into small pieces, and digested with trypsin. LC MS/MS analysis of the peptide 92–115 modified with C-SO_2_H (b_20_ ion [M+2H]^2+^ with *m*/*z* 1016.1^2+^) (C) and C-SO_3_H (b_20_ ion [M+2H]^2+^ with *m*/*z* 1024.1^2+^) (D), respectively. The final concentration of SOD1 was 10 µM.

It has been proposed that aberrant copper activity may be occurring within SOD1 at an alternative binding, and Cys-111, located as a free cysteine on the protein’s surface, has been identified as a potential copper ligand [9], [16], [17], [24], [28], [44], [45]. Therefore, we want to know the role of Cys-111 in A4V oxidation. A4V, wild-type SOD1, and C111S were incubated with 20–400 µM Cu^2+^ for 3 h and then subjected to 13.5% SDS-PAGE. As shown in Fig. 4A, both A4V and wild-type SOD1 had an additional upper shifted band when samples were incubated with 50–400 µM Cu^2+^. Oxidized A4V (upper shifted band) was clearly observed when A4V was incubated with 50–400 µM Cu^2+^ for 3 h (Fig. 4A, lane a), and oxidized wild-type SOD1 (upper shifted band) was faintly observed when wild-type SOD1 was incubated with 100–400 µM Cu^2+^ for 3 h (Fig. 4A, lane b). In contrast, no oxidized band was observed when C111S was incubated with 50–400 µM Cu^2+^ for 3 h (Fig. 4A, lane c), suggesting that Cys-111 should be a primary target for oxidative modification of human SOD1 by 50–400 µM Cu^2+^. Similar upper shifted bands have been observed when wild-type SOD1 is incubated with 1/5/10 mM H_2_O_2_ [25], [28] or 1 mM Cu^2+^ [28]. It has been demonstrated that Cys-111 is a primary target for oxidative modification of wild-type SOD1 by H_2_O_2_ [25], [28], but it is unclear whether Cys-111 is a primary target for oxidative modification of pathological human SOD1 mutants by Cu^2+^. We thus employed LC MS/MS to firmly demonstrate that Cys-111 is a primary target for oxidative modification of human SOD1 by Cu^2+^.

A4V was incubated with 50 µM Cu^2+^ for 3 h and the samples were separated by Coomassie Blue R250-stained SDS-PAGE. The upper shifted bands on SDS-PAGE were clipped out, cut into small pieces, alkylated with iodoacetamide, and digested with trypsin. The sample was then used for LC MS/MS analysis. Fig. 4B shows schematic representation of peptide 92–115 (DGVADVSIEDSVISLSGDHCIIGR) obtained from fragmentations of A4V by LC MS/MS analysis. Fig. 4C shows LC MS/MS analysis of the peptide 92–115 modified with C-SO_2_H (b_20_ ion [M+2H]^2+^ with *m*/*z* 1016.1^2+^), and Fig. 4D displays that of the peptide 92–115 modified with C-SO_3_H (b_20_ ion [M+2H]^2+^ with *m*/*z* 1024.1^2+^). Oxidative modification of Cys-111 (cysteine di- and tri-oxidative modification) by Cu^2+^ was clearly observed in A4V (Fig. 4C and 4D). Taken together, our mass spectrometry and SDS-PAGE data demonstrate that Cys-111 is a primary target for oxidative modification of human SOD1 by cupric ions under copper-mediated oxidative conditions.

### Cupric Ions Not Only Triggered the Oligomerization of Oxidized A4V, but also Triggered the Aggregation of Non-oxidized form of A4V

**Figure 5:**
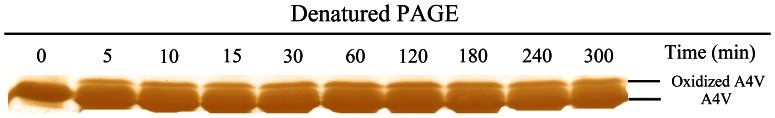
Cu^2+^-induced oxidation of A4V at various time points. 13.5% SDS-PAGE of A4V incubated with 200 µM Cu^2+^ in 20 mM Tris-HCl buffer (pH 7.4) up to 5 h. Oxidized A4V (upper shifted band) was clearly observed when A4V was incubated with 200 µM Cu^2+^ for 5–300 min. The incubation times (from left to right) were 0, 5, 10, 15, 30, 60, 120, 180, 240, and 300 min, respectively. Gels were stained with silver. The final concentration of SOD1 was 10 µM.

Cu^2+^-induced oxidation was further detected by SDS-PAGE (Fig. 5). Fig. 5 shows 13.5% SDS-PAGE of A4V incubated with cupric ions. Oxidized A4V (upper shifted band) was clearly observed when 10 µM A4V was incubated with 200 µM Cu^2+^ for 5-300 min (Fig. 5), indicating that A4V could be oxidized by cupric ions at the early stage. Furthermore, the amount of A4V aggregates detected by light scattering assays (Fig. 1) and AFM (Fig. 3) appears to be gradually increased by increasing incubation time, while the amount of oxidized A4V almost did not change (Fig. 5). The above results indicated that free cupric ions in solution not only induced the oxidation of A4V at the early stage and triggered the oligomerization and aggregation of oxidized A4V under copper-mediated oxidative conditions, but also triggered the aggregation of non-oxidized form of such a pathogenic mutant.

**Figure 6:**
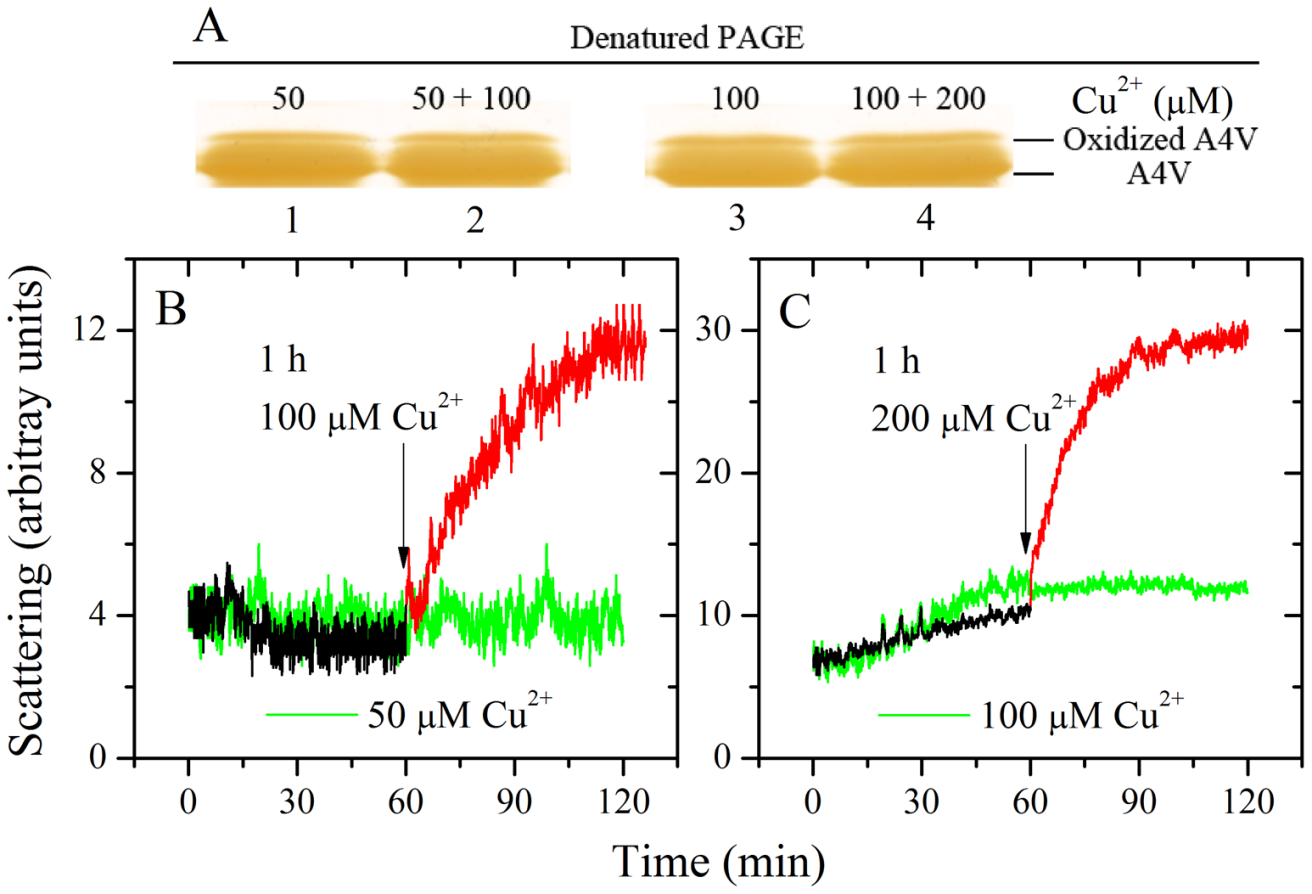
Effect of Cu^2+^ on A4V aggregation at various time points. 13.5% SDS-PAGE of 10 µM A4V incubated with 50 µM Cu^2+^ in 20 mM Tris-HCl buffer (pH 7.4) for 1 h (lane 1) and then incubated with 150 µM Cu^2+^ for 1 h (lane 2), and that of 10 µM A4V incubated with 100 µM Cu^2+^ for 1 h (lane 3) and then incubated with 300 µM Cu^2+^ for 1 h (lane 4) (A). Gels were stained with silver. 10 µM A4V was incubated 50 µM Cu^2+^ in 20 mM Tris-HCl buffer (pH 7.4) (black curves) and then added another 100 µM Cu^2+^ (red curves) (B). 10 µM A4V was incubated 100 µM Cu^2+^ in 20 mM Tris-HCl buffer (pH 7.4) (black curves) and then added another 200 µM Cu^2+^ (red curves) (C). The time of addition is marked by black arrows and the curves are compared with 10 µM A4V incubated with 50 µM Cu^2+^ (green curves) (B) and 10 µM A4V incubated with 100 µM Cu^2+^ (green curves) (C), respectively. Aggregation was measured by 90^o^ light scattering at 37°C.

To get a better understanding about the effect of Cu^2+^ on A4V aggregation, we performed light scattering assays at various time points. Fig. 6A shows 13.5% SDS-PAGE of 10 µM A4V after incubation with 50 µM Cu^2+^ for 1 h (lane 1) and further incubated with an additional 100 µM Cu^2+^ for 1 h (lane 2), and that of 10 µM A4V after incubation with 100 µM Cu^2+^ for 1 h (lane 3) and further incubated with an additional 200 µM Cu^2+^ for 1 h (lane 4). Fig. 6B displays the time-course for the aggregation of 10 µM A4V induced by 50 µM Cu^2+^ and followed with another addition of 100 µM Cu^2+^. Fig. 6C shows the time-course for the aggregation of 10 µM A4V after treatment with 100 µM Cu^2+^ and then treated with another 200 µM Cu^2+^. Both aggregates formed by oxidized A4V (upper shifted band in Fig. 6A) and those formed by the non-oxidized form of apo A4V were clearly observed in both cases (Fig. 6B and 6C). As shown in Fig. 6A and 6B, A4V aggregation was not observed in the presence of 50 µM Cu^2+^ in the time scales used, but the addition of 50 µM Cu^2+^ did induce the oxidation of A4V, and the presence of 150 µM Cu^2+^ (50 µM Cu^2+^ plus 100 µM Cu^2+^) not only induced the oxidation of A4V but also triggered A4V aggregation. As shown in Fig. 6A and 6C, the addition of 100 µM Cu^2+^ not only induce the oxidation of A4V but also triggered A4V aggregation, and subsequent addition of 200 µM Cu^2+^ further accelerated A4V aggregation, suggesting that Cu^2+^ plays a key role in the mechanism of A4V aggregation.

### Cupric Ions also Induced the Oxidation and Triggered the Aggregation of A4V Preequilibrated with a Stoichiometric Amount of Zn^2+^

**Figure 7:**
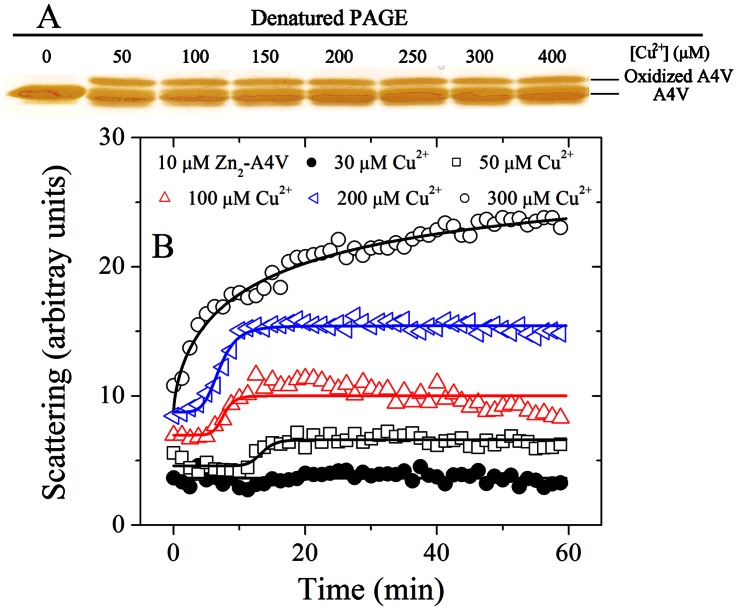
Effects of Cu^2+^ on the oxidation and aggregation of pathological human SOD1 mutant preequilibrated with a stoichiometric amount of Zn^2+^. 13.5% SDS-PAGE of Zn_2_-A4V incubated with 0–400 µM Cu^2+^ in 20 mM Tris-HCl buffer (pH 7.4) for 3 h at 37°C (A). The copper concentrations (from left to right) were 0, 50, 100, 150, 200, 250, 300, and 400 µM, respectively. Gels were stained with silver. Oxidized A4V (upper shifted band) was clearly observed when Zn_2_-A4V was incubated with 50–400 µM Cu^2+^ for 3 h. Time-course for the aggregation of Zn_2_-A4V incubated with 30–300 µM Cu^2+^ in 20 mM Tris-HCl buffer (pH 7.4) (B). The final concentration of SOD1 was 10 µM. The copper concentrations were 30 µM (solid circle), 50 µM (open square), 100 µM (open triangle), 200 µM (left open triangle), and 300 µM (open circle), respectively. The excitation and emission wavelengths both were 350 nm and the slit bands were 10 nm and 3 nm, respectively. The empirical Hill equation was fitted to the data and the solid lines represented the best fit. Aggregation was measured by 90^o^ light scattering at 37°C.

The above results were obtained in the absence of a stoichiometric amount of Zn^2+^. Since zinc binding has strong effects on SOD1 folding, stability, and misfolding [46]–[48], we further take into account the presence of zinc bound to SOD1 variants, a variable that should be addressed. Fig. 7 shows the effects of Cu^2+^ on the oxidation and aggregation of A4V SOD1 preequilibrated with a stoichiometric amount of Zn^2+^. As can be seen from Figs. 4A and 7A, both apo A4V and Zn_2_-A4V had an additional upper shifted band when samples were incubated with 50–400 µM Cu^2+^. Oxidized A4V (upper shifted band) was clearly observed when Zn_2_-A4V was incubated with 50–400 µM Cu^2+^ for 3 h (Fig. 7A). Fig. 7B shows the time-course for the aggregation of 10 µM Zn_2_-A4V incubated with 30–300 µM Cu^2+^ at physiological pH. To our surprise, the addition of 50–300 µM Cu^2+^ triggered the aggregation of A4V preequilibrated with a stoichiometric amount of Zn^2+^ (Fig. 7B), although addition of 2.0 equiv of Zn^2+^ per apo-SOD1 dimer strongly enhances thermostability of the protein [48]. When the concentration of Cu^2+^ went higher, the kinetic curves of Zn_2_-A4V aggregation went up gradually but with a lag phase, and the lag time became shorter gradually (Fig. 7B). Therefore, as revealed by 90° light scattering and SDS-PAGE, free cupric ions in solution not only induced the oxidation of Zn_2_-A4V and trigger the aggregation of oxidized Zn_2_-A4V under copper-mediated oxidative conditions, but also trigger the aggregation of non-oxidized form of Zn_2_-A4V (Fig. 7).

### Oxidation of A4V on Cys-111 by Hydrogen Peroxide also Involved C-SO_2_H and C-SO_3_H

**Figure 8:**
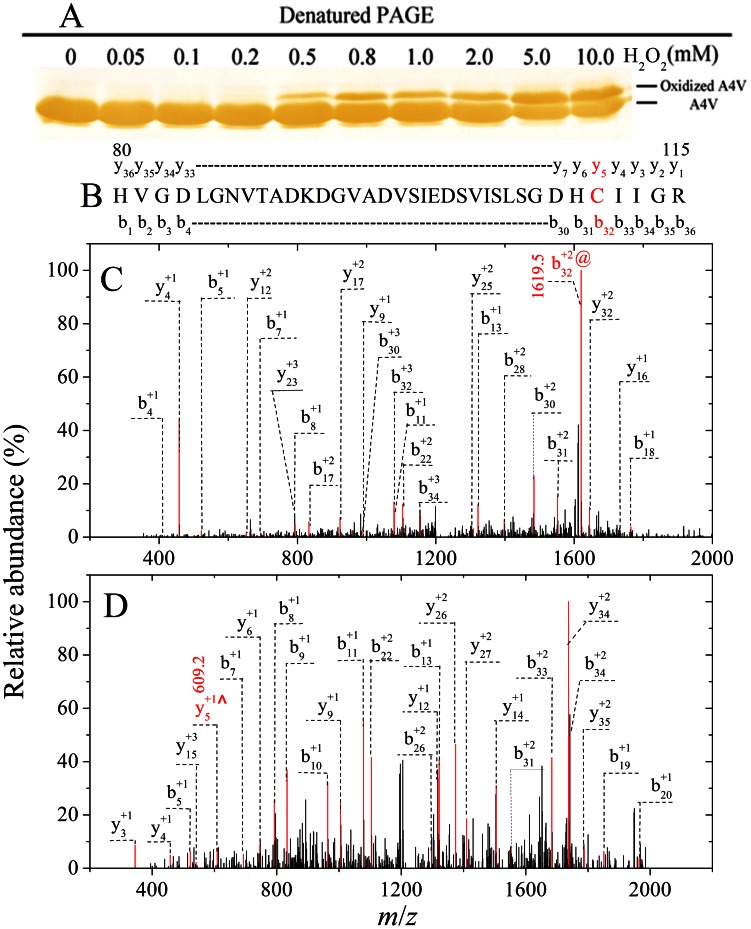
Mass spectrometry and SDS-PAGE demonstrate that Cys-111 is a primary target for oxidative modification of A4V by hydrogen peroxide. 13.5% SDS-PAGE of A4V incubated with 0–10.0 mM H_2_O_2_ in 20 mM Tris-HCl buffer (pH 7.4) for 3 h at 37°C (A). The H_2_O_2_ concentrations (from left to right) were 0, 0.05, 0.1, 0.2, 0.5, 0.8, 1.0, 2.0, 5.0, and 10.0 mM, respectively. Gels were stained with silver. Oxidized A4V (upper shifted band) was clearly observed when A4V was incubated with 0.5–10.0 mM H_2_O_2_ for 3 h (A). Schematic representation of peptide 80–115 obtained from fragmentations of A4V by LC MS/MS analysis (B). The upper shifted bands of A4V incubated with 5.0 mM H_2_O_2_ for 3 h on Coomassie Blue R250-stained SDS-PAGE were clipped out, cut into small pieces, and digested with trypsin. LC MS/MS analysis of the peptide 80–115 modified with C-SO_2_H (b_32_ ion [M+2H]^2+^ with *m*/*z* 1619.5^2+^) (C) and C-SO_3_H (y_5_ ion [M+H]^1+^ with *m*/*z* 609.2^+^) (D), respectively. The final concentration of SOD1 was 10 µM.

Because wild-type SOD1 can be oxidized by both H_2_O_2_ and Cu^2+^ [25], [28], we performed a full comparison between copper and hydrogen peroxide mediated oxidation of A4V SOD1 in order to distinguish between these two oxidative environments. A4V was incubated with 0–10.0 mM H_2_O_2_ for 3 h and then subjected to 13.5% SDS-PAGE. As shown in Fig. 8A, A4V SOD1 had an additional upper shifted band when samples were incubated with 0.5–10.0 mM H_2_O_2_, and oxidized A4V (upper shifted band) was clearly observed under such conditions. Clearly, Cys-111 is a primary target for oxidative modification of A4V by Cu^2+^ (Fig. 4), but it is unclear whether Cys-111 is a primary target for oxidative modification of such a pathological human SOD1 mutant by H_2_O_2_. We thus employed LC MS/MS to demonstrate that Cys-111 is a primary target for oxidative modification of A4V by H_2_O_2_.

A4V was incubated with 5.0 mM H_2_O_2_ for 3 h and the samples were separated by Coomassie Blue R250-stained SDS-PAGE. The upper shifted bands on SDS-PAGE were clipped out, cut into small pieces, alkylated with iodoacetamide, and digested with trypsin. The sample was then used for LC MS/MS analysis. Fig. 8B shows schematic representation of peptide 80–115 (HVGDLGNVTADKDGVADVSIEDSVI SLSGDHCIIGR) obtained from fragmentations of A4V by LC MS/MS analysis. Fig. 8C shows LC MS/MS analysis of the peptide 80–115 modified with C-SO_2_H (b_32_ ion [M+2H]^2+^ with *m*/*z* 1619.5^2+^), and Fig. 8D displays that of the peptide 80-115 modified with C-SO_3_H (y_5_ ion [M+H]^1+^ with *m*/*z* 609.2^+^). Oxidative modification of Cys-111 (cysteine di- and tri-oxidative modification) by H_2_O_2_ was clearly observed in A4V (Fig. 8C and 8D). Taken together, our mass spectrometry and SDS-PAGE data demonstrate that Cys-111 is also a primary target for oxidative modification of A4V by hydrogen peroxide.

### Cupric Ions also Triggered the Aggregation of Oxidized Wild-type SOD1

**Figure 9:**
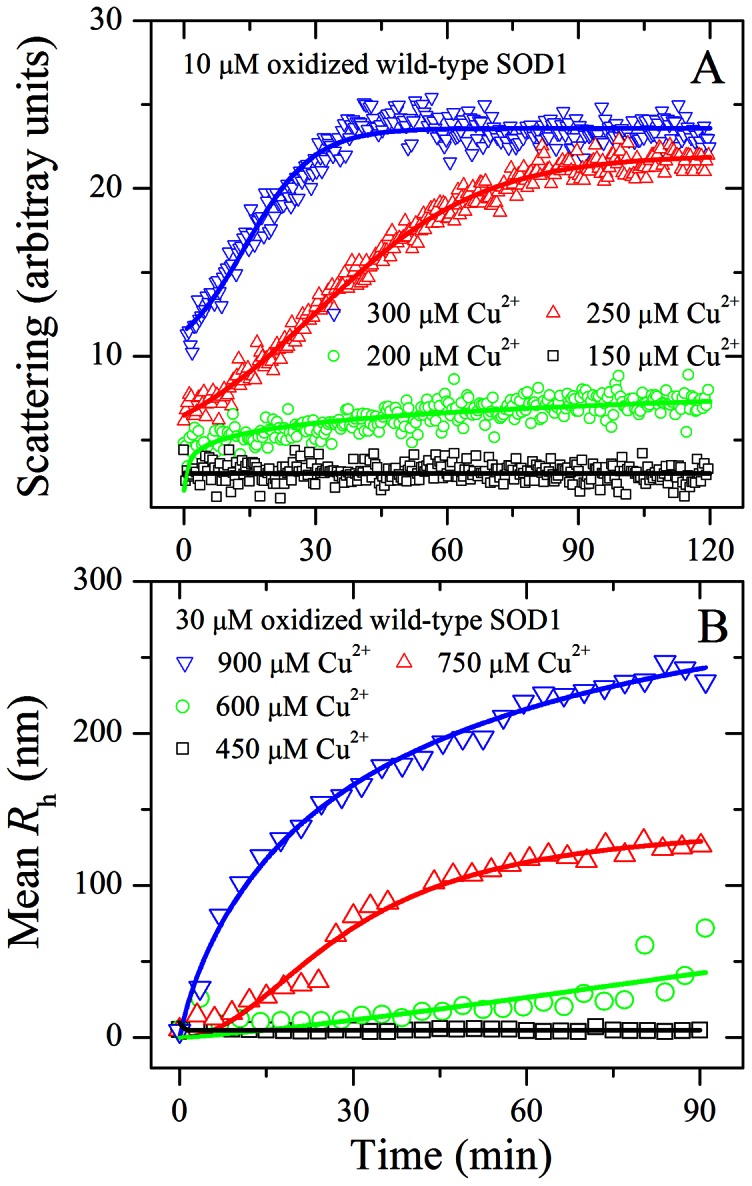
Effects of Cu^2+^ on oxidized wild-type SOD1 aggregation. Time-course for the aggregation of wild-type SOD1 oxidized by hydrogen peroxide incubated with 150–300 µM Cu^2+^ (A) or 450–900 µM Cu^2+^ (B) in 20 mM Tris-HCl buffer (pH 7.4). To obtain oxidized wild-type SOD1, wild-type SOD1 were treated with 5 mM hydrogen peroxide for 2 h. The final concentration of SOD1 was 10 (A) and 30 (B) µM, respectively. The copper concentrations were 150 µM (open square), 200 µM (open circle), 250 µM (open triangle), and 300 µM (inverted open triangle), respectively. The excitation and emission wavelengths both were 350 nm and the slit bands were 10 nm and 3 nm, respectively. The copper concentrations were 450 µM (open square), 600 µM (open circle), 750 µM (open triangle), and 900 µM (inverted open triangle left open triangle), respectively (B). The mean hydrodynamic radius (*R*_h_) was calculated by use of the method of cumulants. The empirical Hill equation was fitted to the data and the solid lines represented the best fit. Aggregation was measured by 90^o^ light scattering at 37°C (A) or dynamic light scattering (B).

Because we found that ALS-associated SOD1 mutant A4V was induced to aggregate by cupric ions under copper-mediated oxidative conditions, we want to know the role of Cu^2+^ in aggregation of oxidized wild-type SOD1 which exists in sporadic ALS [25], [49]. To obtain oxidized wild-type SOD1, wild-type SOD1 were treated with 5 mM hydrogen peroxide for 2 h. Fig. 9A shows the time-course for aggregation of 10 µM oxidized wild-type SOD1 incubated with 150–300 µM Cu^2+^. As shown in Figs. 9A and 1, similar to A4V, the addition of 300 µM Cu^2+^ did trigger the aggregation of oxidized wild-type SOD1 monitored by 90^o^ light scattering, but aggregation of non-oxidized form of wild-type SOD1 was not observed in the presence of 300 µM Cu^2+^ in the time scales used. When the concentration of Cu^2+^ went higher, the kinetic curves of the oxidized wild-type SOD1 went up gradually, almost without a lag phase (Fig. 9A). As shown in Figs. 9A and 1, however, the addition of 150 µM Cu^2+^ did trigger the aggregation of A4V, but aggregation of the oxidized wild-type SOD1 was not observed in the presence of 150 µM Cu^2+^ in the time scales used. Fitting the aggregation kinetic data of the oxidized wild-type SOD1 with the empirical Hill equation (Fig. 9A) and the seventh order polynomial equation (Fig. S3A–3C) gave *LS*(∞) and *k*_max_ values, respectively, which reflect the final quantity and the elongation phase of aggregation of the oxidized wild-type SOD1, respectively. The corresponding kinetic parameters are summarized in Table 1. As shown in Table 1, at the same concentration of Cu^2+^, the aggregation of the oxidized wild-type SOD1 was much faster than that of unoxidized wild-type SOD1 but remarkably slower than that of A4V. Furthermore, we performed parallel experiments using dynamic light scattering technique that provides quantitative estimates of the properties of oxidized SOD1 aggregate species. Fig. 9B shows the time-course for the aggregation of 30 µM oxidized wild-type SOD1 incubated with 450–900 µM Cu^2+^ at physiological pH. As shown in Fig. 9B, the addition of 600–900 µM Cu^2+^ did trigger 30 µM oxidized wild-type SOD1 aggregation monitored by dynamic light scattering. Similar to Fig. 9A, the kinetic curves of oxidized wild-type SOD1 aggregation went up gradually with the increase of the concentration of Cu^2+^, almost without a lag phase (Fig. 9B). As can be seen from Fig. 9B, the average hydrodynamic radii increased with increasing both the concentration of Cu^2+^ and the incubation time. Taken together, our light scattering data demonstrated that excess cupric ions triggered the aggregation of oxidized wild-type SOD1 much faster than its non-oxidized form under copper-mediated oxidative conditions, in a way similar to A4V. Our data also suggest that Cu^2+^ plays an important role in the mechanism of aggregation of wild-type SOD1 oxidized by hydrogen peroxide.

Analyses of aggregation kinetics shown in Figs. 1A and 9A were fitted in two different ways: using an empirical Hill equation or using some sort of polynomial fit, which are clearly explained in the Materials and Methods section. A visual inspection of the kinetic transients in Fig. 1 suggests the presence of at least two kinetic phases, with very different time scales. Actually, a comparison of the kinetic data in Fig. 1 (up to 60 min) and Fig. 3 (AFM results, up to 10 h) suggest that the slow phase suggested by light scattering results may represent the growth of large granular aggregates. To check whether SOD1 aggregation follows the biphasic first-order mechanism, a double exponential model [32] was used to analyze the kinetic data in Figs. 1A and 9A, and the kinetic parameters for the aggregation were thus determined:
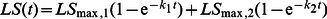
(5)where *LS*_max,1_ and *LS*_max,2_ represent the maximal light scattering intensities for the fast phase and the slow phase, respectively, and *k*_1_ and *k*_2_ are the rate constants for the fast phase and the slow phase respectively. The corresponding kinetic parameters are also summarized in Table 1. As shown in Table 1, the value of *LS*_max,1_+ *LS*_max,2_ for SOD1 aggregation could be determined precisely at each copper concentration. However, the rate constants for the fast phase and the slow phase of either oxidized wild-type SOD1 aggregation at each copper concentration or the aggregation of A4V in the presence of 100/150 µM Cu^2+^ were not determined because the 90° light scattering data in the present conditions could be fitted to such a double exponential model with a huge error (Table 1). Both rate constants (*k*_1_ and *k*_2_) for A4V aggregation in the presence of 200–300 µM Cu^2+^ were quite good (Table 1), suggesting that A4V aggregation could follow the biphasic first-order mechanism under such conditions.

### Cu^2+^ Bound to Oxidized Wild-type SOD1 in a Way Similar to A4V

**Figure 10:**
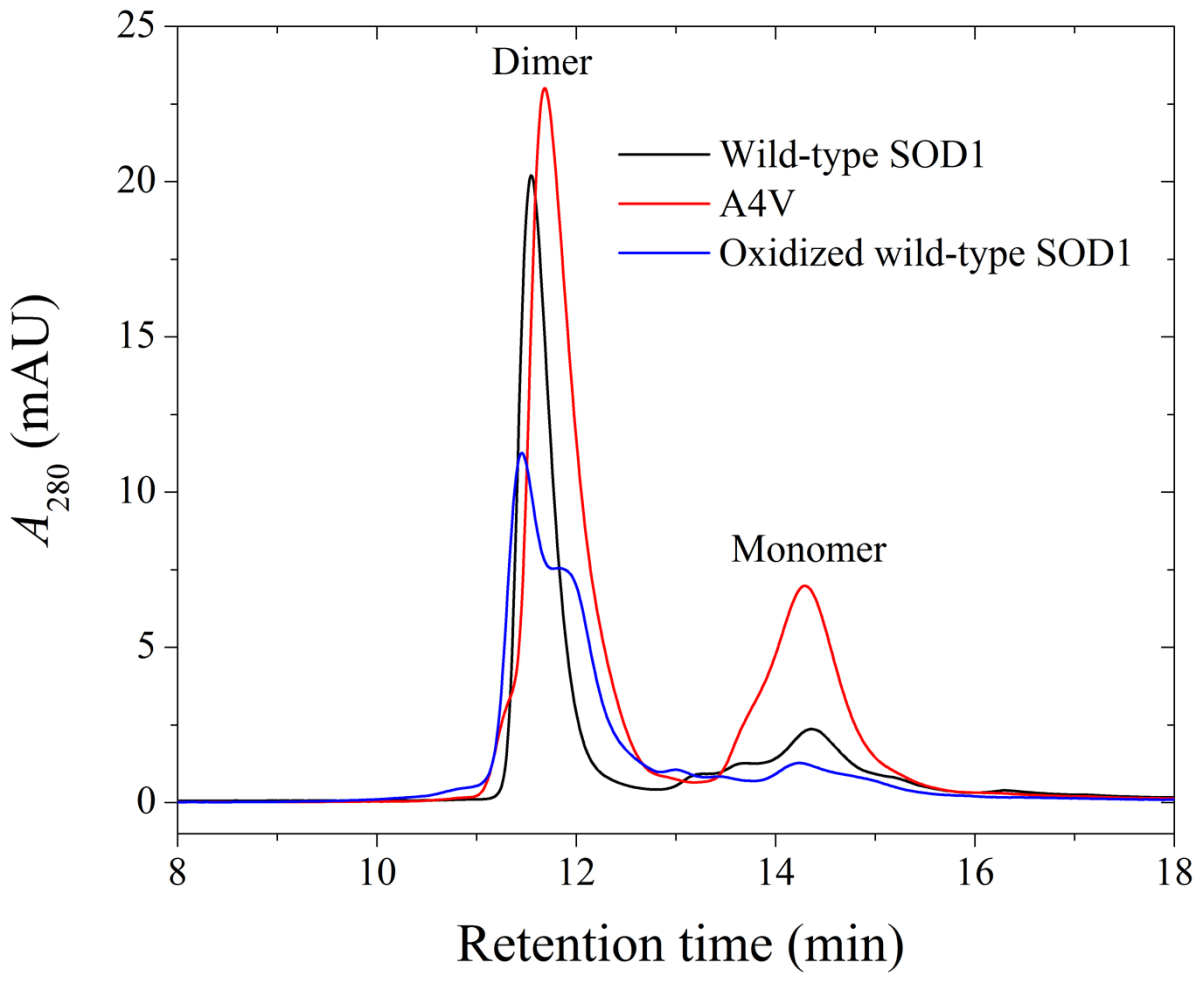
Size-exclusion chromatography measurements of wild-type SOD1, A4V, and oxidized wild-type SOD1. Wild-type SOD1 (black line), A4V (red line), and wild-type SOD1 oxidized by hydrogen peroxide (blue line) samples (20–30 µM when injected) in 100 mM phosphate buffer (pH 6.7) containing 100 mM Na_2_SO_4_ and 0.05% NaN_3_. The absorbance was measured at 280 nm.

Here we found that apo-SOD1 consisted of dimer (major) and monomer (minor) at pH 6.7, and thus used size-exclusion chromatography (SEC) to separate them. Fig. 10 shows SEC measurements of wild-type SOD1, A4V, and wild-type SOD1 oxidized by hydrogen peroxide under such slightly acidic conditions. As shown in Fig. 10, the retention times corresponding to monomeric SOD1 were all ∼14.3 min, and those corresponding to dimeric wild-type SOD1, dimeric A4V, and dimeric oxidized wild-type SOD1 were ∼11.5 min, ∼11.8 min, and ∼11.5/∼11.8 min, respectively. Apo wild-type SOD1 eluted predominantly as a dimer (Fig. 10), similar to the previous reports [50], [51]. In contrast, apo A4V was an equilibrium mixture of dimer and monomer (Fig. 10). It should be pointed out that dimeric wild-type SOD1 eluted earlier than dimeric A4V, indicating that the dimeric conformations of wild-type SOD1 and A4V were a little different from each other. The elution profile of dimeric oxidized wild-type SOD1 (Fig. 10) showed two peaks eluted at ∼11.5 and ∼11.8 min, one is similar to dimeric wild-type SOD1 and one is similar to dimeric A4V, indicating that the dimeric conformations of oxidized wild-type SOD1 and A4V were similar in part.

**Figure 11:**
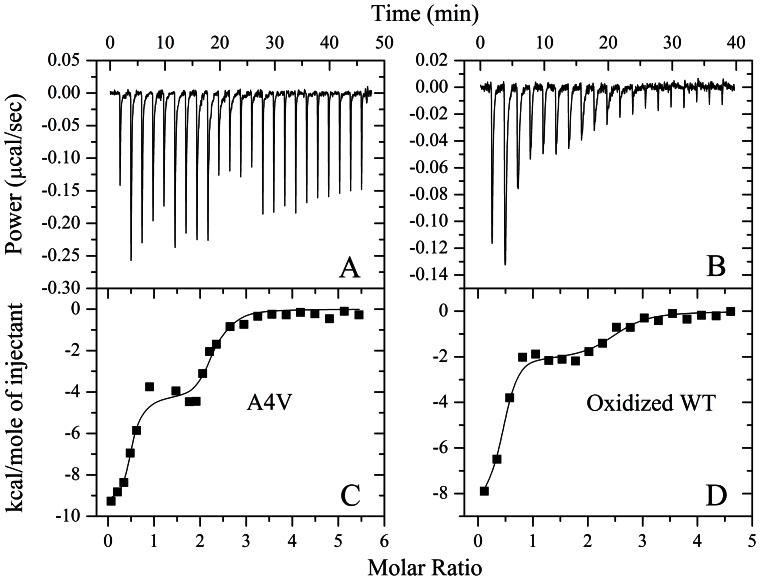
ITC profiles for the binding of Cu^2+^ to A4V and oxidized wild-type SOD1 at 25.0°C. The top panels represent the raw data for sequential injections of 300 µM Cu^2+^ into 10.7 µM A4V (A) and sequential 2-µl injections of 250 µM Cu^2+^ into 10.7 µM wild-type SOD1 oxidized by hydrogen peroxide (WT) (B) in 20 mM Tris-HCl buffer (pH 7.4), respectively. The first 4 injections of 1 µl were followed by 4 injections of 2 µl, and then 4 injections of 1 µl and 11 injections of 2 µl (A). The bottom panels (C and D) show the plots of the heat evolved (kcal) per mole of Cu^2+^ added, corrected for the heat of Cu^2+^ dilution, against the molar ratio of Cu^2+^ to SOD1. The data (solid squares) were best fitted to a model of two independent sets of binding sites and the solid lines represented the best fit.

So far only one group has studied the binding of Cu^2+^ to wild-type SOD1 by using ITC [52], [53], although ITC has been widely used to investigate the binding of metal ions to proteins [33], [36], [48], [52]–[56]. In this study, we employed ITC to investigate the binding of Cu^2+^ to ALS-associated SOD1 mutant A4V, compared with wild-type SOD1 oxidized by hydrogen peroxide. ITC profiles for the binding of Cu^2+^ to A4V and oxidized wild-type SOD1 at 25.0°C are shown in Fig. 11. Fig. 11, A and B, representatively shows raw ITC curves resulting from the injections of Cu^2+^ into a solution of A4V and the oxidized wild-type SOD1. The titration curves show that copper binding to A4V and the oxidized wild-type SOD1 is exothermic, resulting in negative peaks in the plots of power versus time. The bottom panels in Fig. 11 show the plots of the heat evolved per mole of Cu^2+^ added, corrected for the heat of Cu^2+^ dilution, against the molar ratio of Cu^2+^ to A4V (Fig. 11C) and the oxidized wild-type SOD1 (Fig. 11D). The calorimetric data were best fitted to a model of two independent sets of binding sites as described by several papers [52]–[55]. The thermodynamic parameters for the binding of Cu^2+^ to the apo form of human SOD1 are summarized in Table 2. As shown in Table 2, A4V possessed two sets of independent binding sites for Cu^2+^: a moderate-affinity site (5.37×10^6^) and a high-affinity site (5.47×10^8^). Similarly, Cu^2+^ bound to the oxidized wild-type SOD1 *via* two independent binding sites, one with a moderate affinity (2.23×10^6^) and one with a high affinity (2.04×10^8^) (Table 2). The ITC-derived stoichiometry for the two sites indicated that about 2 and 1 molecules of Cu^2+^ bound to the moderate- and high-affinity sites respectively (Table 2 and Fig. 11). The binding reactions for both A4V and the oxidized wild-type SOD1 were driven by large favorable increases in entropy in combination with moderately favorable enthalpy decreases (Table 2). Clearly, Cu^2+^ bound to wild-type SOD1 oxidized by hydrogen peroxide in a way similar to A4V, triggering the aggregation of such an oxidized form. We thus suggest that Cu^2+^ plays a key role in the mechanism of aggregation of both A4V and the oxidized wild-type SOD1.

**Table 2:** Thermodynamic parameters for the binding of Cu^2+^ to the apo form of human SOD1 as determined by ITC at 25.0^o^C.

SOD1	Site	*K*_b_ (M^−1^)	*n*	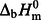 (kcal mol^−1^)	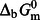 (kcal mol^−1^)	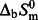 (cal mol^−1^ K^−1^)
V	Site 1	(5.37±3.54) ×10^6^	1.76±0.06	−4.35±0.37	−9.18±0.39	16.2±2.5
A4V	Site 2	(5.47±5.08) ×10^8^	0.447±0.034	−9.31±0.46	−11.92±0.55	8.75±3.39
Oxidized wild-type SOD1	Site 1	(2.23±1.50) ×10^6^	2.05±0.10	−2.10±0.19	−8.66±0.40	22.0±2.0
Oxidized wild-type SOD1	Site 2	(2.04±1.49) ×10^8^	0.359±0.022	−8.33±0.45	−11.33±0.43	10.1±3.0

Thermodynamic parameters, *K*_b_, 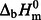 and *n*, were determined using a model of two independent sets of binding sites. The standard molar binding free energy (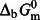) and the standard molar binding entropy (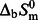) for the binding reaction were calculated using Equations 3 and 4, respectively. The buffer used was 20 mM Tris-HCl buffer (pH 7.4). Errors shown are standard errors of the mean.

## Discussion

ALS, a fatal degenerative disease of motor neurons, is partly caused by the mutations and aggregation of SOD1 [1]. Copper is one of the most important transition metals in the brain but are toxic in excess [10]-[13]. It has been reported that the loss of zinc from SOD1 results in the remaining copper in SOD1 to become extremely toxic to motor neurons in ALS [57]. Because SOD1 is a well-known copper-binding protein present at higher concentration and longer half-life in motor neurons than other cells, there are some environmental factors existing within motor neurons, such as aberrant copper biochemistry [9], [15], that induce SOD1 oxidation and trigger SOD1 aggregation specifically in this cell type [38], [58]. The long life span of SOD1 in motor neurons will increase the chances of oxidative modification by copper and/or hydrogen peroxide; one possible byproduct of oxidative modification is induction of SOD1 aggregation [38]. In the present study, we investigated and compared the impact of cupric ions on the aggregation of pathological human SOD1 mutant A4V and wild-type SOD1 oxidized by hydrogen peroxide. We found that Cu^2+^ bound to both A4V and the oxidized wild-type SOD1 *via* two independent binding sites, one with a moderate affinity and one with a high affinity. We demonstrated that excess cupric ions not only induced the oxidation of A4V and triggered the aggregation of oxidized A4V under copper-mediated oxidative conditions, but also triggered the aggregation of unoxidized A4V and the oxidized wild-type SOD1. All the results above demonstrate that A4V and the oxidized wild-type SOD1 are much more easily aggregated in the presence of cupric ions under copper-mediated oxidative conditions than wild-type SOD1, and it is possible that the pathogenic properties of A4V and oxidized wild-type SOD1 are at least in part due to this aggregation. Thus, it seems reasonable to speculate that familial ALS and sporadic ALS share common mechanisms of copper ion regulation of oxidative modification and aggregation of SOD1, which might lead to a common, or at least partly overlapping, pathogenic mechanisms.

In the present study, we described SEC studies on the oligomeric status of A4V and wild-type SOD1 variants, showing a higher monomeric fraction in the A4V mutant. Moreover, these SEC analyses also suggest that the dimer-monomer equilibrium is slow, since two peaks are separated. Our results might be very relevant to explain the higher propensity of the A4V mutant to aggregate, since it is known that SOD1 monomers show enhanced aggregation propensity [59], thus explaining the higher aggregation propensity of A4V.

In this paper, we considered that within the experimental uncertainty (which is large, in fact), Cu^2+^ bound to A4V and the oxidized wild-type SOD1 with similar properties. However, visual inspection of titration profiles (Fig. 11) and fitting parameters (Table 2) suggests some differences in binding thermodynamic properties, for example, the binding constants for Cu^2+^-A4V interaction are two-fold larger than those of Cu^2+^ bound to the oxidized wild-type SOD1. These differences could potentially influence the copper concentration dependence of SOD1 (A4V and wild-type SOD1 oxidized by H_2_O_2_) aggregation kinetics (Figs. 1 and 9 and Table 1). For instance, the concentration of Cu^2+^ that is required to trigger aggregation of the oxidized wild-type SOD1 is two-fold larger than that required to trigger A4V aggregation. Moreover, the role of the two different sets of sites in SOD1 needs to be discussed. It has been reported that 2.0 equiv of Cu^2+^ per apo-SOD1 dimer bind first to the copper site, giving a characteristic d-d transition band at 670 nm, and additional Cu^2+^ then binds to the zinc site, giving an additional overlapping absorption band with a maximum at 810 nm [44]. ITC determination of binding constants for Cu^2+^ to apo-SOD1 at pH 5 also shows that copper binds apparently to all four metal-binding sites [52]. Based on our ITC data and the reported results [44], [52], we concluded that Cu^2+^ could bind to a Zn^2+^ binding site of SOD1 (A4V and wild-type SOD1 oxidized by H_2_O_2_).

SOD1 aggregates observed in ALS patients and transgenic mouse models have been described as granular aggregates and granule-coated fibrillar aggregates [60]-[63]. Our AFM results demonstrated that excess cupric ions triggered A4V oligomerization, inducing such a pathogenic mutant to form abundant granular aggregates under copper-mediated oxidative conditions. Thus, the structural characteristics of A4V aggregates induced by copper resemble those observed in ALS [60]-[62]. Furthermore, neither red shift of the maximum absorbance of Congo red nor enhancement of thioflavin T intensity was observed for such A4V aggregates (Fig. S4), resembling those observed in ALS [60], [61]. Taken together, we suggest that Cu^2+^-mediated oxidative chemistry partly underlies the pathogenesis of familial ALS linked to mutations of SOD1 gene.

Only a small percentage of ALS patients carry an inherited mutation in SOD1 [4], [5], [49], [64], [65], yet some studies have revealed that the SOD1 protein does not work properly even in sporadic ALS patients without a known mutation in the gene [9], [16], [25], [28], [49], [64], [65]. It has been concluded that wild-type SOD1 acquires binding and toxic properties of ALS-linked mutant forms through oxidation by hydrogen peroxide [64]. It has been reported that oxidized wild-type SOD1, an aberrant wild-type SOD1 species, does exist in motor neurons from sporadic ALS patients [25]. Very recently, the Pasinelli lab [49] has demonstrated that wild-type SOD1 is over-oxidized in certain disease-fighting white blood cells in sporadic ALS patients with bulbar onset. This over-oxidized wild-type SOD1 acquires toxic properties of pathological SOD1 mutants [49]. Using a conformation-specific antibody C4F6 that detects misfolded SOD1, Bosco and co-workers [25] have found that oxidized wild-type SOD1 and pathogenic mutant SOD1 share a conformational epitope that is not present in normal wild-type SOD1. In this paper, we found that Cu^2+^ bound to wild-type SOD1 oxidized by hydrogen peroxide in a way similar to ALS-associated SOD1 mutant A4V and thus triggered the aggregation of such an oxidized form, supporting the above conclusions. These findings suggest that aberrant copper biochemistry is one of the causing factors in triggering the aggregation of oxidized wild-type SOD1, and that oxidized wild-type SOD1 may be a contributor to motor neuronal death in sporadic ALS [9].

The mechanism by which familial ALS mutants induce ALS disease remains to be elucidated. Enhanced aggregate formation is one of the proposed toxic gain-of-functions exerted by familial ALS mutants, due mainly to the observed precipitates of mutant SOD1 (and wild-type SOD1) in the spinal cord of ALS patients or animals [4]-[9]. However, the report by Son et al. [66] showing that over-expression of both G93A and CCS in mice leads to accelerated neurological deficits with no detectable SOD1 aggregates, indicates that aggregate formation is not absolutely required for familial ALS disease. Interestingly, our observed Cu^2+^-mediated oxidation that subsequently leads to aggregate formation is in accordance with the notion that familial ALS mutants and/or Cu^2+^-mediated free radical generation may be the initial event that leads to SOD1 mutant-linked protein aggregation, and mitochondrial pathology [67], and the report showing early mRNA oxidation occurs in ALS [68]. In order to mimic conditions in the cell to some extent, we preequilibrated A4V with a stoichiometric amount of Zn^2+^, and found excess cupric ions still induced the oxidation and triggered the aggregation of Zn_2_-A4V. Although our results were obtained under non-physiological conditions, such as Cu^2+^ binding in the absence of CCS (required for proper Cu^2+^ binding to the catalytic site of SOD1) [14], nevertheless, our findings are intriguing and will yield mechanistic insight into how free radical-mediated SOD1 mutants aggregate under pathological conditions observed in a number of neurodegenerative diseases.

It should be pointed out that even in the absence of CCS, SOD1 still retains 15-20% copper-mediated activity and cupric ions can still bind to SOD1 [17], [22], [69]. At physiological pH cupric ions form two complexes with Tris [70]. We demonstrated that Cys-111 of 10 µM A4V was oxidized by 50 µM Cu^2+^-Tris complexes and that the theoretical minimum concentration of Cu^2+^-Tris complex to induce A4V aggregation under such conditions was 40 µM. The results from ITC showed that A4V recruited about 3 mol eq of Cu^2+^-Tris complexes with a moderate binding affinity and a high binding affinity at physiological pH. Similar binding constants for Cu^2+^-apo-SOD1 interaction in 10 mM pivalate buffer have been reported [52], suggesting that our ITC results should not be modified when using other buffers. Therefore 10 µM “free” Cu^2+^-Tris complexes could oxidize Cys-111 of 10 µM A4V and triggered A4V aggregation. It has been estimated that independent copper binding still represents a substantial concentration of intracellular Cu^2+^ (10 µM) in SOD1-overexpressing transgenic mice [69], suggesting a possibility of 10 µM Cu^2+^ to induce the oxidation and aggregation of ALS-associated SOD1 mutants *in vivo* even in the absence of CCS. In fact it has been found that mutant SOD1, but not wild-type SOD1, shifts intracellular copper homeostasis toward copper accumulation in the spinal cord during the progression of familial ALS [71].

There are four cysteine residues, Cys-6, Cys-57, Cys-111, and Cys-146, in a subunit of human SOD1. Among them, Cys-57 and Cys-146 form an intramolecular disulfide bond that maintains the rigid structure and enzymatic activity of SOD1, whereas Cys-6 and Cys-111 are present as free cysteines. Cys-6 is deeply buried in the protein molecule and less accessible by substrates and other molecules, whereas Cys-111, located on the surface of SOD1 near the dimer interface, is the most solvent-exposed cysteine in human SOD1 [9], [28], [44], [45]. Cys-111 in SOD1 is known to undergo oxidation to its sulfinic and sulfonic derivatives [25], [28]. SOD1 with oxidized Cy-111 appears to be correlated with the formation of SOD1 aggregates leading to sporadic ALS [9], [16], [25], [28], [45], [72]. This oxidative modification of Cys-111 promotes the formation of disulfide bond-independent aggregation of SOD1 [72] and plays an important role in oxidative damage to human SOD1 [9], [17], [29], [46]. In this manuscript, we used light scattering, AFM, mass spectrometric, and isothermal titration calorimetric methods to show that excess cupric ions mediates oxidation of Cys-111 and leads to aggregate formation of ALS-associated SOD1 mutant A4V and wild-type SOD1 oxidized by hydrogen peroxide under copper-mediated oxidative conditions. Very recently, the Borchelt lab [24] has described the lack of ALS in mice that express a variant of human SOD1 in which residues that coordinate the binding of copper and zinc have been mutated. This novel variant encodes three disease-causing and four experimental mutations that ultimately eliminate all histidines involved in the binding of metals; and includes one disease-causing and one experimental mutation that eliminate secondary metal binding at Cys-6 and Cys-111. The combined effect of these mutations produces a protein that is unstable but does not aggregate on its own, is not toxic, and does not induce ALS when co-expressed with high levels of wild-type SOD1 [24]. All the results above suggest that oxidative modification of Cys-111 plays a significant role in the onset and development of ALS by increasing the formation of neurotoxic aggregates of human SOD1.

**Figure 12:**
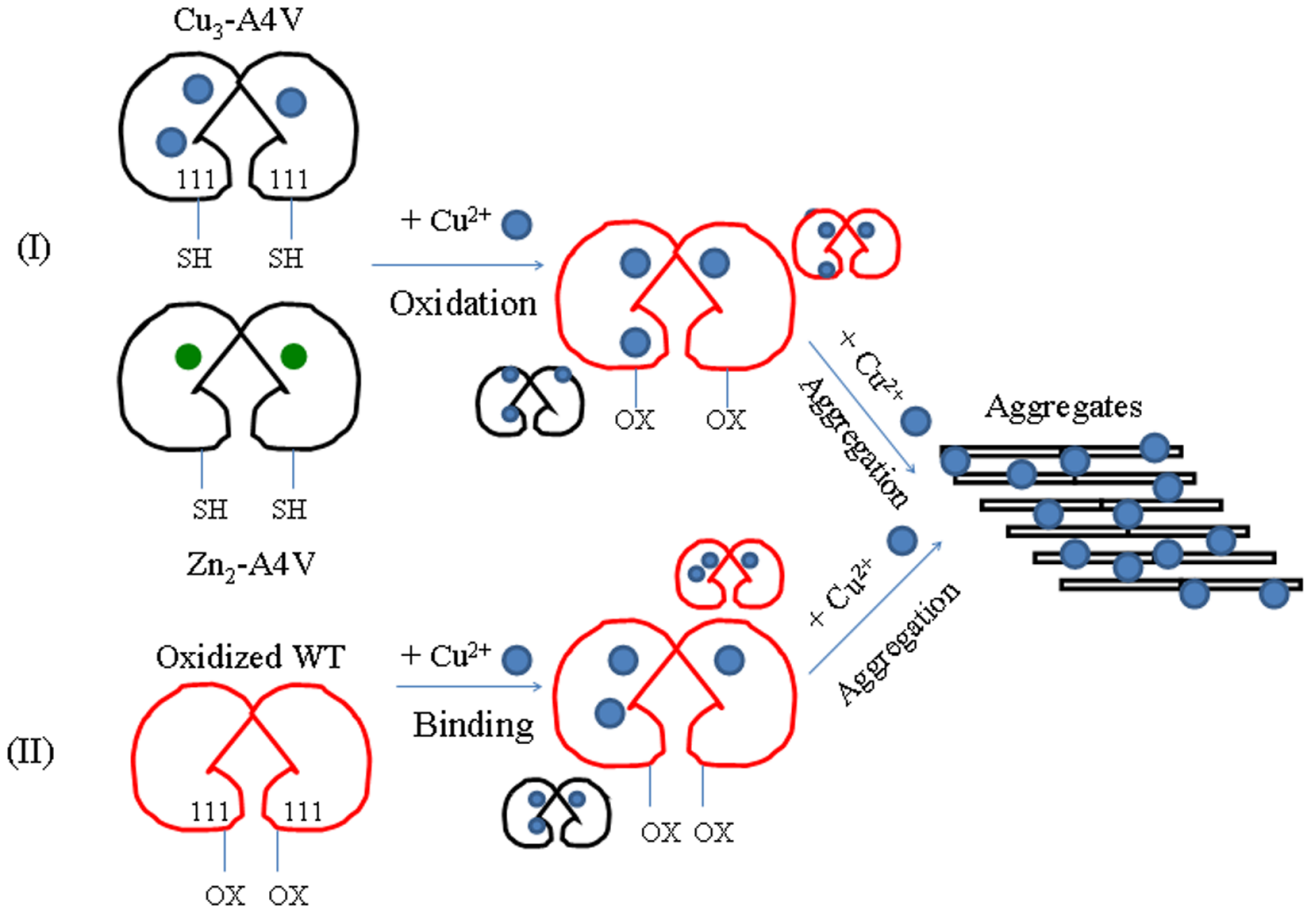
Hypothetical models for copper inducing the oxidation and triggering the aggregation of human SOD1 under copper mediated oxidative conditions. In model (I), the first step is the oxidation of either Cu_3_-A4V (black dimer with three blue circles) or Zn_2_-A4V (black dimer with two green circles) induced by free copper in solution, forming oxidized A4V (red dimer). In model (II), the first step is the binding of Cu^2+^ (blue circle) to wild-type SOD1 (WT) oxidized by hydrogen peroxide (red dimer), forming a 3∶1 complex.

Based on our kinetic and thermodynamic data and the reported results [9], [16], [17], [24], [28], [44], [45], we propose valuable hypothetical models for copper inducing the oxidation and triggering the aggregation of human SOD1 under copper-mediated oxidative conditions (Fig. 12). In model (I), the first step is the oxidation of either Cu_3_-A4V or Zn_2_-A4V induced by free copper in solution, forming oxidized A4V. Since aggregation occurs in this mutant at comparatively high copper concentrations (and comparatively long time scales for metal binding), it is expected that at least a large fraction of protein undergoing oxidation of Cys-111 by free copper would have the copper binding sites already occupied. The second step is that excess cupric ions not only trigger the aggregation of oxidized A4V under copper-mediated oxidative conditions, but also trigger the aggregation of non-oxidized form of such a pathogenic mutant. In model (II), the first step is the binding of Cu^2+^ to wild-type SOD1 oxidized by hydrogen peroxide, forming a 3∶1 complex. The second step is that excess cupric ions not only trigger the aggregation of the oxidized wild-type SOD1, but also trigger the aggregation of non-oxidized form of wild-type SOD1 (Fig. 12). Clearly, SOD1 mutants could gain new aberrant toxic functions through binding to Cu^2+^, causing their oxidation and aggregation. It has been indicated that the nature of mutant SOD1 toxicity could involve the dysregulation of the copper trafficking pathway, resulting in the disruption of intracellular copper homeostasis [71]. Our data provide a plausible model to explain how pathological SOD1 mutants aggregate in ALS-affected motor neurons with the disruption of copper homeostasis, and will be helpful to the understanding of the role of aberrant copper biochemistry in the pathogenesis of ALS.

## Supporting Information

Figure S1 **The dependence of 90^o^ light scattering intensity changes for SOD1 aggregation at 37**°**C on the concentration of Cu^2+^.** Time-course for the aggregation of A4V incubated with 100–300 µM Cu^2+^ in 20 mM Tris-HCl buffer (pH 7.4). The final concentration of SOD1 was 10 µM. The copper concentrations were 100 µM (A), 150 µM (B), 200 µM (C), 250 µM (D), and 300 µM (E), respectively. A seventh order polynomial was fitted to all data (open circles) from replicates at each Cu^2+^ concentration. The kinetic curves were analyzed as described under “Materials and Methods”.
(DOC)

Figure S2 **Size distribution by intensity for Cu^2+^-induced SOD1 aggregation at various time points.** The hydrodynamic radius (*R*_h_) distribution for the aggregation of 30 µM A4V incubated with 600 µM Cu^2+^ in 20 mM Tris-HCl buffer (pH 7.4). The incubation time was 0 min (A), 2.58 min (B), 15.48 min (C), and 59.34 min (D), respectively. Aggregation was measured by dynamic light scattering.
(DOC)

Figure S3 **The dependence of 90^o^ light scattering intensity changes for oxidized SOD1 aggregation at 37°C on the concentration of Cu^2+^.** Time-course for the aggregation of wild-type SOD1 oxidized by hydrogen peroxide incubated with 200–300 µM Cu^2+^ in 20 mM Tris-HCl buffer (pH 7.4). The final concentration of SOD1 was 10 µM. The copper concentrations were 200 µM (A), 250 µM (B), and 300 µM (C), respectively. A seventh order polynomial was fitted to all data (open circles) from replicates at each Cu^2+^ concentration. The kinetic curves were analyzed as described under “Materials and Methods”.
(DOC)

Figure S4 **A4V aggregates induced by Cu^2+^ did not possess typical dye binding properties of amyloid.** Absorbance data are shown for aggregation of 30 µM A4V incubated with 600 µM Cu^2+^ in 20 mM Tris-HCl buffer (pH 7.4) in the presence of 50 µM Congo red at 6 h (A). The difference spectra (d) were obtained by subtracting the absorbance spectra of A4V aggregates alone (c) and Congo red alone (a) from those of A4V aggregates+Congo red (b). Time-course for the aggregation of A4V incubated with 0–600 µM Cu^2+^ in 20 mM Tris-HCl buffer (pH 7.4) up to 24 h, monitored by thioflavin T (ThT) fluorescence (B). The final concentrations of A4V and ThT were both 30 µM. The copper concentrations were 0 µM (open square), 300 µM (solid circle), and 600 µM (solid triangle), respectively. The fluorescence of ThT was excited at 440 nm with a slit-width of 10 nm and the emission was measured at 480 nm with a slit-width of 5 nm on an LS-55 luminescence spectrometer (PerkinElmer Life Sciences, Shelton, CT). Congo red binding assays and ThT binding assays were carried out at 37**°**C.
(DOC)
