# Supplementary material for: Cupric Ions Induce the Oxidation and Trigger the Aggregation of Human Superoxide Dismutase 1
Source: PLoS One. 2013 Jun 3;8(6):e65287. doi: 10.1371/journal.pone.0065287 (PMC3670862; doi:10.1371/journal.pone.0065287)
Supplement: Supplementary file 2 [file pone.0065287.s002.doc]

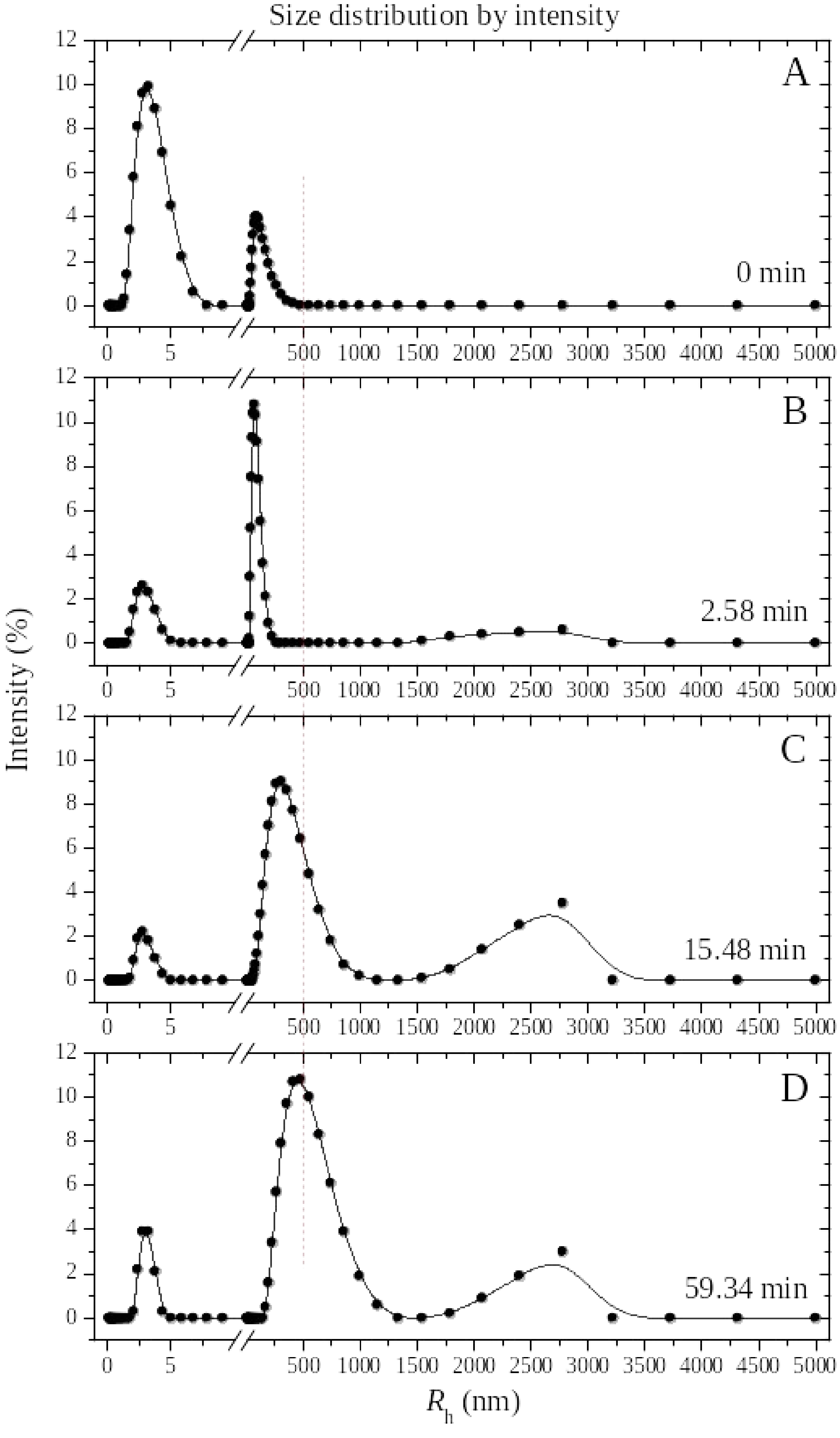

Size distribution by intensity
Intensity (%)
A
0 min
B
2.58 min
C
15.48 min
D
59.34 min
0
2
4
6
8
10
12
0
5
500
1000
1500
2000
2500
3000
3500
4000
4500
5000
R_h (nm)
