# Supplementary material for: Cupric Ions Induce the Oxidation and Trigger the Aggregation of Human Superoxide Dismutase 1
Source: PLoS One. 2013 Jun 3;8(6):e65287. doi: 10.1371/journal.pone.0065287 (PMC3670862; doi:10.1371/journal.pone.0065287)
Supplement: Supplementary file 3 [file pone.0065287.s003.doc]

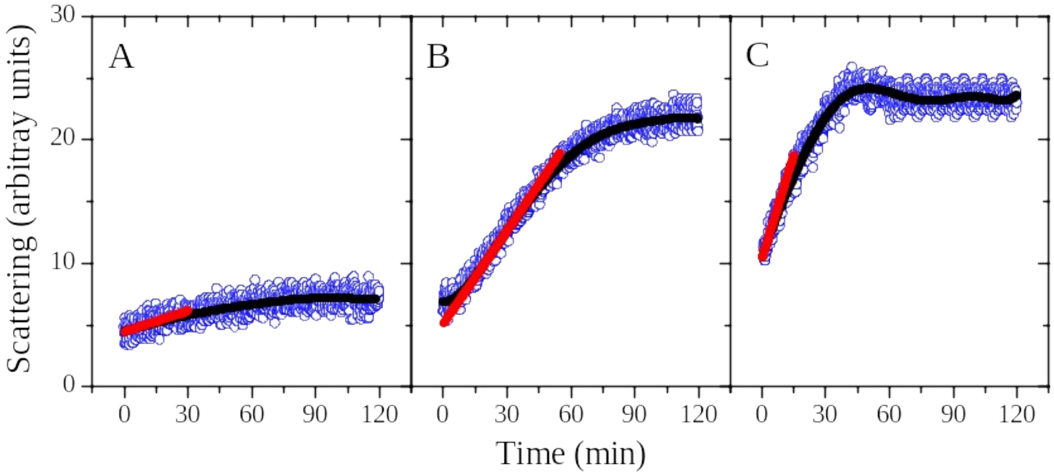
A
B
C
Scattering (arbitray units)
Time (min)
0
10
20
30
0
30
60
90
120
