# Supplementary material for: Cupric Ions Induce the Oxidation and Trigger the Aggregation of Human Superoxide Dismutase 1
Source: PLoS One. 2013 Jun 3;8(6):e65287. doi: 10.1371/journal.pone.0065287 (PMC3670862; doi:10.1371/journal.pone.0065287)
Supplement: Supplementary file 4 [file pone.0065287.s004.doc]

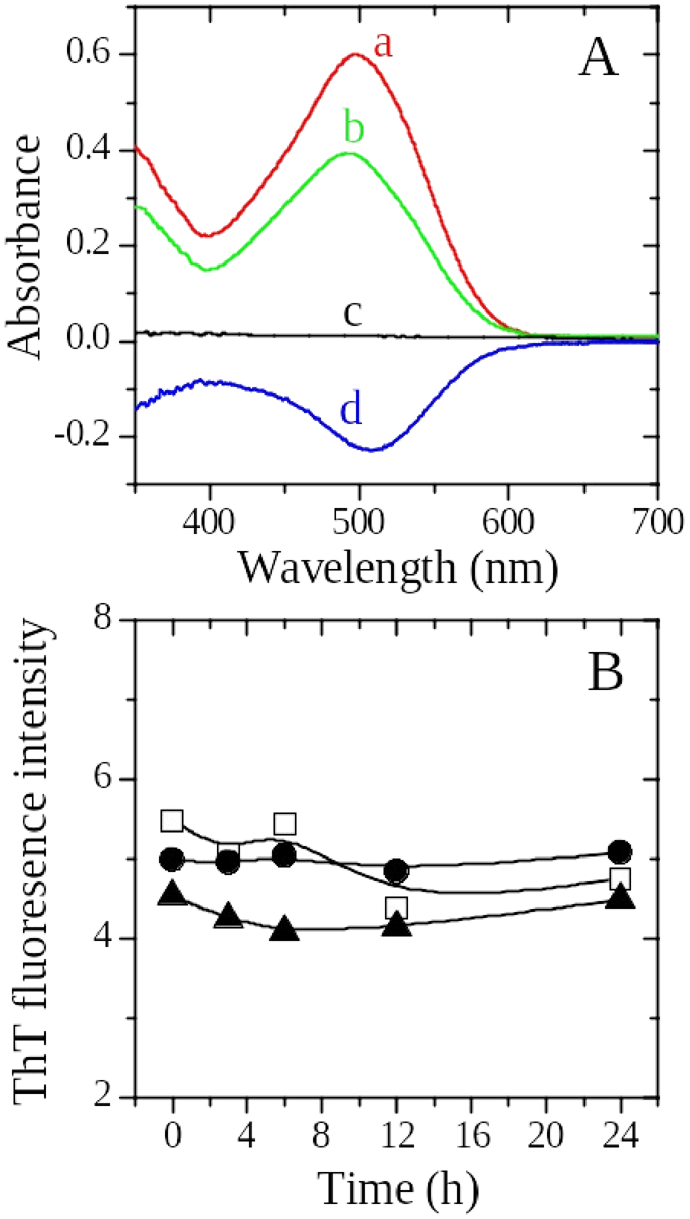
A
a
b
c
d
Absorbance
0.6
0.4
0.2
0.0
-0.2
400
500
600
700
Wavelength (nm)
B
ThT fluoresence intensity
8
6
4
2
0
4
8
12
16
20
24
Time (h)
